# A Transdiagnostic Community-Based Mental Health Treatment for Comorbid Disorders: Development and Outcomes of a Randomized Controlled Trial among Burmese Refugees in Thailand

**DOI:** 10.1371/journal.pmed.1001757

**Published:** 2014-11-11

**Authors:** Paul Bolton, Catherine Lee, Emily E. Haroz, Laura Murray, Shannon Dorsey, Courtland Robinson, Ana M. Ugueto, Judith Bass

**Affiliations:** 1 Department of International Health, Johns Hopkins Bloomberg School of Public Health, Baltimore, Maryland, United States of America; 2 Department of Mental Health, Johns Hopkins Bloomberg School of Public Health, Baltimore, Maryland, United States of America; 3 Department of Psychology, University of Washington, Seattle, Washington, United States of America; Massachusetts General Hospital, United States of America

## Abstract

**Background:**

Existing studies of mental health interventions in low-resource settings have employed highly structured interventions delivered by non-professionals that typically do not vary by client. Given high comorbidity among mental health problems and implementation challenges with scaling up multiple structured evidence-based treatments (EBTs), a transdiagnostic treatment could provide an additional option for approaching community-based treatment of mental health problems. Our objective was to test such an approach specifically designed for flexible treatments of varying and comorbid disorders among trauma survivors in a low-resource setting.

**Methods and Findings:**

We conducted a single-blinded, wait-list randomized controlled trial of a newly developed transdiagnostic psychotherapy, Common Elements Treatment Approach (CETA), for low-resource settings, compared with wait-list control (WLC). CETA was delivered by lay workers to Burmese survivors of imprisonment, torture, and related traumas, with flexibility based on client presentation. Eligible participants reported trauma exposure and met severity criteria for depression and/or posttraumatic stress (PTS). Participants were randomly assigned to CETA (*n* = 182) or WLC (*n* = 165). Outcomes were assessed by interviewers blinded to participant allocation using locally adapted standard measures of depression and PTS (primary outcomes) and functional impairment, anxiety symptoms, aggression, and alcohol use (secondary outcomes). Primary analysis was intent-to-treat (*n* = 347), including 73 participants lost to follow-up. CETA participants experienced significantly greater reductions of baseline symptoms across all outcomes with the exception of alcohol use (alcohol use analysis was confined to problem drinkers). The difference in mean change from pre-intervention to post-intervention between intervention and control groups was −0.49 (95% CI: −0.59, −0.40) for depression, −0.43 (95% CI: −0.51, −0.35) for PTS, −0.42 (95% CI: −0.58, −0.27) for functional impairment, −0.48 (95% CI: −0.61, −0.34) for anxiety, −0.24 (95% CI: −0.34, −0.15) for aggression, and −0.03 (95% CI: −0.44, 0.50) for alcohol use. This corresponds to a 77% reduction in mean baseline depression score among CETA participants compared to a 40% reduction among controls, with respective values for the other outcomes of 76% and 41% for anxiety, 75% and 37% for PTS, 67% and 22% for functional impairment, and 71% and 32% for aggression. Effect sizes (Cohen's *d*) were large for depression (*d* = 1.16) and PTS (*d* = 1.19); moderate for impaired function (*d* = 0.63), anxiety (*d* = 0.79), and aggression (*d* = 0.58); and none for alcohol use. There were no adverse events. Limitations of the study include the lack of long-term follow-up, non-blinding of service providers and participants, and no placebo or active comparison intervention.

**Conclusions:**

CETA provided by lay counselors was highly effective across disorders among trauma survivors compared to WLCs. These results support the further development and testing of transdiagnostic approaches as possible treatment options alongside existing EBTs.

**Trial registration:**

ClinicalTrials.gov NCT01459068

*Please see later in the article for the Editors' Summary*

## Abstract 2

In a randomized controlled trial, Paul Bolton and colleagues investigate whether a transdiagnostic community-based intervention is effective for improving mental health symptoms among Burmese refugees in Thailand.

*Please see later in the article for the Editors' Summary*

## Editors' Summary

**Background:**

Worldwide, one in four people will experience a mental health disorder at some time during their life. Although many evidence-based treatments (EBTs), most involving some sort of cognitive behavioral therapy (talking therapies that help people manage their mental health problems by changing the way they think and behave), are now available, many people with mental health disorders never receive any treatment for their condition. The situation is particularly bad for people living in low-resource settings, where a delivery model for EBTs based on referral to mental health professionals is problematic given that mental health professionals are scarce. To facilitate widespread access to mental health care among poor and/or rural populations in low-resource settings, EBTs need to be deliverable at the primary or community level by non-professionals. Moreover, because there is a large burden of trauma-related mental health disorders in low-resource settings and because trauma increases the risk of multiple mental health problems, treatment options that address comorbid (coexisting) mental health problems in low-resource settings are badly needed.

**Why Was This Study Done?:**

One possible solution to the problem of delivering EBTs for comorbid mental health disorders in low-resource settings is “transdiagnostic” treatment. Many mental health EBTs for different disorders share common components. Transdiagnostic treatments recognize these facts and apply these common components to a range of disorders rather than creating a different structured treatment for each diagnosis. The Common Elements Treatment Approach (CETA), for example, trains counselors in a range of components that are similar across EBTs and teaches counselors how to choose components, their order, and dose, based on their client's problems. This flexible approach, which was designed for delivery by non-professional providers in low-resource settings, provides counselors with the skills needed to treat depression, anxiety, and posttraumatic stress—three trauma-related mental health disorders. In this randomized controlled trial, the researchers investigate the use of CETA among Burmese refugees living in Thailand, many of whom are survivors of decades-long harsh military rule in Myanmar. A randomized controlled trial compares the outcomes of individuals chosen to receive different interventions through the play of chance.

**What Did the Researchers Do and Find?:**

The researchers assigned Burmese survivors or witnesses of imprisonment, torture, and related traumas who met symptom criteria for significant depression and/or posttraumatic stress to either the CETA or wait-list control arm of their trial. Lay counselors treated the participants in the CETA arm by delivering CETA components—for example, “psychoeducation” (which teaches clients that their symptoms are normal and experienced by many people) and “cognitive coping” (which helps clients understand that how they think about an event can impact their feelings and behavior)—chosen to reflect the client's priority problems at presentation. Participants in the control arm received regular calls from the trial coordinator to check on their safety but no other intervention. Participants in the CETA arm experienced greater reductions of baseline symptoms of depression, posttraumatic stress, anxiety, and aggression than participants in the control arm. For example, there was a 77% reduction in the average depression score from before the intervention to after the intervention among participants in the CETA arm, but only a 40% reduction in the depression score among participants in the control arm. Importantly, the effect size of CETA (a statistical measure that quantifies the importance of the difference between two groups) was large for depression and posttraumatic stress, the primary outcomes of the trial. That is, compared to no treatment, CETA had a large effect on the symptoms of depression and posttraumatic stress experienced by the trial participants.

**What Do These Findings Mean?:**

These findings suggest that, among Burmese survivors and witnesses of torture and other trauma living in Thailand, CETA delivered by lay counselors was a highly effective treatment for comorbid mental disorders compared to no treatment (the wait-list control). These findings may not be generalizable to other low-resource settings, they provide no information about long-term outcomes, and they do not identify which aspects of CETA were responsible for symptom improvement or explain the improvements seen among the control participants. Given that the study compared CETA to no treatment rather than a placebo (dummy) or active comparison intervention, it is not possible to conclude that CETA works better that existing treatments. Nevertheless, these findings support the continued development and assessment of transdiagnostic approaches for the treatment of mental health disorders in low-resource settings where treatment access and comorbid mental health disorders are important challenges.

**Additional Information:**

Please access these websites via the online version of this summary at http://dx.doi.org/10.1371/journal.pmed.1001757.

- The World Health Organization provides background information about mental health
- The US National Institute of Mental Health provides information about a range of mental health disorders and about cognitive behavioral therapy
- The UK National Health Service Choices website has information about cognitive behavioral therapy, including some personal stories and links to other related mental health resources on the Choices website
- A short introduction to transdiagnosis and CETA written by one of the trial authors is available
- Information about this trial is available on the ClinicalTrials.gov website
- The UN Refugee Agency provides information about Burmese (Myanmar) refugees in Thailand

## Introduction

### Background

Violence and other traumas increase the risk of multiple mental health problems including symptoms of posttraumatic stress (PTS), depression, and anxiety. Studies of mental health programs addressing these problems in humanitarian settings range from trials of psychodynamic therapy to stress and coping skills training to more structured therapies including cognitive behavioral therapy (CBT), interpersonal psychotherapy (IPT), Narrative Exposure Therapy, Cognitive Processing Therapy, and testimony therapy [1]. A number of randomized controlled trials (RCTs) have now been completed on some of these interventions in lower resource settings using a task-sharing approach in which providers with limited mental health training or education provided the intervention [2],[3]. Most trials have shown positive clinical outcomes in comparison to active (e.g., [4],[5]) and wait-list (e.g., [6]) comparison conditions. Although usually highly structured to focus on a single disorder such as PTS or depression (with specific guidelines for the content of each session, number of sessions, and sequencing of session content), several treatments have also been found effective for multiple disorders and other psychosocial problems.

Given the diversity of mental disorders that can arise from trauma (depression, anxiety, substance abuse, panic, psychosis, borderline personality, etc.), and the large burden of trauma-related mental disorders in low-resource settings where mental health professionals are scarce, there remains a need for treatment options that can both address a range of mental health problems and be delivered by individuals with little mental health training. One of the strengths of existing evidence-based treatments (EBTs) is their highly structured approach, which facilitates their use by non-professional workers. We wondered whether such workers could also use a less structured, more flexible transdiagnostic approach in which they would individualize treatment plans based on client presentations (e.g., comorbidity, most pressing current problem). If successful, this type of approach could provide an additional community-based treatment option, particularly in areas where there are diverse presentations and where training in multiple EBTs is not an option. This study was designed as an early test of whether a transdiagnostic approach could be successfully implemented by non-professional workers.

### Current Study

The objective of the study described here was to test a transdiagnostic treatment developed for comorbid presentations of depression, anxiety, and trauma symptoms among trauma survivors in a low-resource setting. This version of a transdiagnostic treatment, the Common Elements Treatment Approach (CETA), is based on existing transdiagnostic manuals [7],[8] and is designed specifically for delivery by non-professional providers in low-resource settings [9]. To date, transdiagnostic treatments have been tested only in high-income countries (HICs) and only when implemented by mental health professionals. To our knowledge, transdiagnostic treatments have not been tested in low-resource settings or with non-professional providers.

### Setting

This trial was conducted among Burmese adults displaced into Thailand. Harsh conditions under decades of military rule, including imprisonment of political prisoners, attacks on ethnic minority groups, forced labor, and widespread forced displacement, resulted in the movement of many from Myanmar (Burma) to neighboring countries, specifically Thailand. Since 1984, 2 million Burmese have sought asylum in Thailand [10], including many survivors of systematic violence, including torture, with elevated depression, anxiety, and PTS symptoms (PTSS) [11]. Most lack documentation, work in unsafe conditions, are underpaid, and are at risk of trafficking and exploitation [12]. The trial was conducted by the Applied Mental Health Research Group (AMHR) (Johns Hopkins University), Burma Border Projects (an international nongovernmental organization [NGO]), and three local service organizations—Assistance Association for Political Prisoners–Burma (AAPP), Mae Tao Clinic (MTC), and Social Action for Women (SAW). Financial support was from the US Agency for International Development Victims of Torture Fund.

Identifying the mental health needs of Burmese adults displaced in Thailand and providing appropriate treatment for these individuals requires exploration and incorporation of culturally significant signs and symptoms of distress in the context of varying social experiences [13]. Using un-adapted standardized assessment tools developed in one culture may not accurately capture the psychological problems of another culture, resulting in a distorted picture of distress and functioning. Similarly, imposing treatment developed in a different culture with no consideration of local appropriateness may be harmful and may increase distrust of nontraditional practices [14]. With virtually no mental health care system in Myanmar [15], individuals rely on traditional means of coping such as talking to family members and friends, sleeping, and singing or playing music [11]. In the current study, careful consideration was given to first investigating and adapting the psychosocial assessment tools for use among Burmese refugees, and then to selecting and adapting the therapeutic treatment. This was done using a research and program development model, DIME (design, implementation, monitoring, and evaluation), that aims to (1) identify and measure local mental health problems through qualitative methods, (2) guide the selection, adaptation, and testing of mental health instruments and interventions, and (3) monitor and evaluate provided services in collaboration with local providers and community organizations. Details of this approach are available online [16].

## Methods

### Ethical Approval

This trial was approved by the Institutional Review Board at Johns Hopkins University, the Johns Hopkins Bloomberg School of Public Health, and a local ethics committee in Mae Sot, Thailand. The local committee was composed of five Burmese members from NGOs and community-based organizations, led by a local physician; all members of the committee were knowledgeable about migrant mental health and human rights issues affecting Burmese refugees. The local committee received translated copies of all study procedures and materials, and provided written approval of the trial.

Recruitment began on August 8, 2011, but the trial was registered at ClinicalTrials.gov on Oct 21, 2011. The discrepancy was due to unfamiliarity with the registration process and miscommunication between team members as to when registration occurred. The first follow-up interviews were conducted November 11, 2011, and therefore trial registration occurred before any outcome data were collected.

### Trial Design

This was a single-site, two-arm (1∶1 allocation), single-blinded, wait-list RCT. It was single-blinded in that interviewers at baseline and follow-up did not know to which study arm the interviewees belonged. Outcomes were assessed by interview using locally adapted instruments. Interviews were conducted at recruitment (baseline) and approximately 4 mo later (the maximum duration of treatment plus 1 mo to allow for delays) for both intervention and control groups.

### Participants

**Figure 1:**
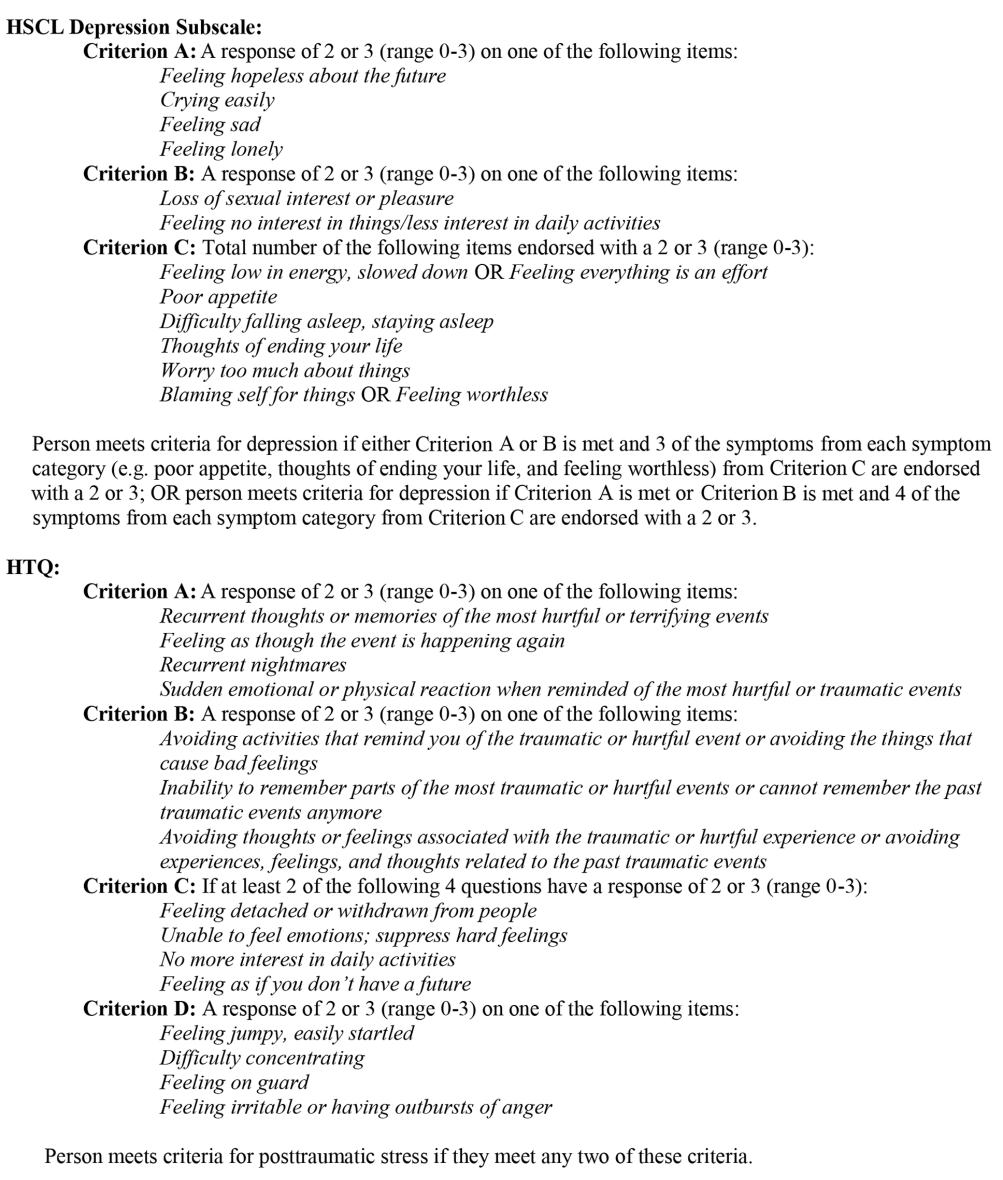
Modified scoring algorithms for HSCL and HTQ.

Participants were Burmese individuals at least 18 y of age. Eligibility criteria were (1) witnessed or experienced a traumatic event and (2) moderate to severe depression and/or PTSS based on locally validated measures. The presence of moderate to severe depression was determined by applying modified versions of previously developed DSM IV–based algorithms to baseline interviews with the Hopkins Symptom Checklist 25 (HSCL-25) [17] and the Harvard Trauma Questionnaire (HTQ) for depression and PTSS, respectively [17]. Prior qualitative research suggested that these algorithms were appropriate for use in this population. The algorithms are shown in Figure 1. The sole exclusion criterion was active psychosis.

### Study Setting

Mae Sot is in northwest Thailand, 5 km from Myanmar. During this study, few mental health services were available to Burmese refugees except counseling at the Burmese-run MTC. Many Burmese reported reluctance to go there (or other public places) for fear of apprehension and deportation by Thai authorities [18].

### Counselors

Counselors and supervisors were staff at one of three local service organizations; qualifications were literacy in Burmese and demonstrated interest in mental health and counseling. All counselors and supervisors were Burmese refugees, were members of the Burmese community in Mae Sot, and shared many cultural, religious, and political experiences with their clients (e.g., imprisonment, forced labor, loss of property). Clinical supervisors had at least a high school education, were bilingual in English and Burmese, and preferably had counseling experience. Counselors (11 female; nine male) ranged in age from 24 to 61 y (mean = 34 y), and some had worked previously as teachers (seven) or health workers (four). Two had prior “general counseling” experience. After training, one supervisor switched to being a counselor because of limited English proficiency. The remaining three supervisors were male, 28–57 y: a doctor, mental health counselor, and a former political prisoner with no counseling experience or advanced degree.

### Intervention

Transdiagnostic mental health interventions capitalize on commonalities and similar components across EBTs (e.g., psychoeducation, cognitive processing) (e.g., [19]). Transdiagnostic approaches involve teaching providers a set of these cross-cutting treatment components, with decision rules and guidelines for which components to use for which presenting problems. Component selection, sequencing, and dosage (e.g., number of sessions per component, number of sessions for the treatment) can be varied based on individual symptom presentation, comorbidity, and most disturbing current problem (e.g., avoidance of trauma triggers or intrusive trauma-related memories). Detailed guidance in these areas is particularly important in low-resource settings when using a task-shifting approach, as providers do not a have mental health background. In HICs, individuals with mental health training may be better able to “flex” manualized EBTs to address individual client needs (e.g., adding a component, extending treatment duration or dosage for a particular component), given their training and experience. In HICs, transdiagnostic approaches have been proposed to address concerns about scale-up of EBTs such as the time and resources needed for training a workforce in multiple EBTs and achieving mastery and fidelity across multiple EBTs [20],[21]. Initial studies of such approaches in HICs have demonstrated effectiveness and a positive response from providers [22]–[24].

CETA is a transdiagnostic treatment approach developed by two authors (L. M. and S. D.) for delivery by lay counselors in low-resource settings with few mental health professionals [9]. Like transdiagnostic approaches developed for HICs, CETA was designed to treat symptoms of common mental health disorders including depression, PTS, and anxiety. Differences between CETA and HIC-based models include the following: (1) fewer elements, (2) simplified language, (3) brief step-by-step guides for each element (1–2 pages), including example quotes of what counselors could say, (4) specific attempts to make the complex concepts of cognitive coping and cognitive restructuring components more accessible to counselors and clients, such as the use of concrete strategies often used in child-focused interventions, and (5) training the provider in element selection, sequencing, and dosing for each client rather than having decision-making done by higher level professionals (who may not be widely available in low-resource settings). For this population, CETA was used to treat depression, anxiety, PTS, aggression, stress due to current life problems, and alcohol abuse, problems that emerged as priorities during a prior qualitative study [25] among individuals similar to those included in this study. CETA as used in this study consisted of nine elements that focused on a torture- and violence-exposed population. These elements are listed and described in Table 1. They include an element to address alcohol abuse: screening and brief intervention (SBI) for alcohol [26]. The SBI element was developed based on motivational interviewing techniques [27] to provide feedback on a personalized assessment of drinking and to assist the client with identifying steps he/she might want to take to reduce or stop drinking. Counselors delivered CETA during weekly 1-h sessions with the client, practicing skills both in and between sessions.

**Table 1:** Elements of CETA.

Component	Brief Description	Inclusion
Engagement (encouraging participation)	Attention to perceived/logistical obstacles to engagement	Provided to all participants
Psychoeducation (introduction)	Program information (duration, content, expectations)Normalization of symptoms/problems	Provided to all participants
Anxiety management (relaxation)	Strategies to reduce physiological tension/stress	Included as optional if client presented with physiological symptoms of anxiety
Behavioral activation (getting active)	Identifying and engaging in pleasurable, mood-boosting activities	Included as optional if client presented with symptoms related to depression
Cognitive coping/restructuring (thinking in a different way—two elements)	Identifying and connecting thoughts, feelings, and behaviorsEvaluating and restructuring thoughts to be more accurate and/or helpful	Provided to all participants
Imaginal gradual exposure (talking about difficult memories)	Facing feared and/or avoided traumatic memories	Provided to all participants because of trauma history
In vivo exposure (live exposure)	Facing innocuous triggers/reminders in the client's environment	Included as optional if client feared and avoided a physical place or thing that was actually safe
Safety (suicide/homicide/danger assessment and planning)	Assessing risk for suicide, homicide, and domestic violenceDeveloping a safety plan	Provided to all participants, used as needed
SBI for alcohol (alcohol intervention)	Utilizing concepts of motivational interviewing to get client buy-in to change drinking	Included as optional if the client had harmful alcohol use (≥8 on AUDIT)

To facilitate acceptance of these skills, counselors tailored the CETA CBT skills to the individual and familial needs of their clients, as well as to the cultural needs of the Burmese community, by using Burmese folktales, personal anecdotes, and local expressions or adages to convey key principles. Cultural modifications also included building on existing strengths (e.g., support of family and community) and existing coping strategies (e.g., meditation, singing songs, having tea with friends) to increase daily functioning. Clients also were encouraged to invite family members and close friends to introductory sessions in order for others to understand the role of counseling and to support the client in the treatment. Clients received CETA in familiar venues where the client felt most comfortable, including the home of the client or counselor, local Burmese-run clinics or community organizations, and secluded outside areas.

Counselors and supervisors were trained using the apprenticeship model [28]. This included a 10-d in-person training followed by practice groups. Practice groups were led by one of three local supervisors, with 3–6 counselors per group practicing CETA elements with each other, supervised by the local supervisor. Following the practice groups, each trainee then treated one pilot client under close supervision by the local supervisors, prior to treating participants in the RCT. Throughout, local supervisors received at least 2 h per week of supervision from the US-based CETA trainers (doctoral-level psychologists) by phone call, Internet call, and/or email. At each stage, the apprenticeship model included feedback loops encouraging local counselors and supervisors to modify delivery of components to increase the fit with the culture and local setting, based on their ongoing experiences. For example, counselors and supervisors could suggest using different ways of stating ideas, or change analogies and examples to improve understanding. Only after successful completion of a pilot case did counselors begin to treat participants in the RCT. If counselors encountered problems such as an inability to complete practice role plays and/or frequently having to repeat elements with the first pilot client because of mistakes, then they took on a second pilot case under close supervision. CETA trainers and local supervisors discussed counselor performance and jointly made decisions about the need for an additional pilot case during weekly Internet calls.

Supervision groups continued throughout the RCT, with each local supervisor meeting with a small group of counselors for 2–4 h per week. Local supervision involved presentation of each and every case, review of client assessments and counselors' treatment plans, review sessions (fidelity monitoring), role plays to practice components, and planning upcoming sessions. All cases were then reported on and discussed with US-based CETA trainers each week, who documented details of each case. Fidelity tracking was done through a multi-tier review approach. Specifically, counselors tracked their own fidelity by following their step sheets and checking off each step on their own step sheets. They also completed a monitoring form for each session, which included documentation of the component delivered and some steps for each component. Supervisors reviewed fidelity during the supervision groups by reviewing the monitoring forms and requiring in-person objective reporting (e.g., “I started with step one, and said we would be working on relaxation exercises because sometimes the client needs skills to reduce stress. Then I taught breathing, describing what we would do, showing the client an example, and had the client practice.”), rather than subjective reporting (e.g., “The client seemed mad and didn't want to work.”), during supervision. This allowed the supervisor to determine which steps within the component were delivered and whether they were delivered correctly. The final and third layer of fidelity checking was completed during weekly Internet calls between supervisors and US-based CETA trainers. Supervisors provided an objective report of the sessions for each case, and the trainers asked questions specific to the steps and the way in which they were completed. If errors within a session occurred (e.g., failure to complete a step, step delivered incorrectly), the supervisor coached the counselor to redo this component or step during the following session.

### Measures

#### Qualitative research for adaptation of measures and intervention

Between August 2010 and February 2011, we conducted a qualitative study to understand the problems and conditions of persons from Myanmar residing in Mae Sot, Thailand, including those specific to persons who had experienced or witnessed torture or other forms of systematic violence. The methodology has been described in detail elsewhere [25]. Briefly, free-listing interviews were conducted among 60 Burmese to identify problems faced by displaced persons in general and problems particularly affecting survivors of torture and systematic violence and their families. Key informant interviews were then conducted among 30 knowledgeable members of the Burmese displaced and migrant community on selected psychosocial problems that emerged from the free-list interviews. Problems were selected from the free lists based on the number of respondents who mentioned the problems, apparent severity, and the possibility that these problems could be addressed by interventions provided at the community level. For each problem, interviewers probed on (1) a description of symptoms and effects, (2) causes, and (3) what people do about the problem or think could or should be done about it.

Information from free-listing and key informant interviews was used to select and adapt standard instruments for local use (and for adaptation of CETA). Instrument adaptation involved the addition of questions on locally relevant symptoms and the use of verbatim qualitative data to appropriately translate concepts.

#### Primary outcome measures

All outcome measures were adapted to the local context and tested during a prior instrument validation study [29]. Adaptations were based on qualitative data. Validation consisted of an exploratory factor analysis, an internal consistency measure (using Cronbach's α) [30], and a combined test-retest/inter-rater reliability measure (using Pearson's product moment correlation coefficient *r*) [31] for each scale used to measure outcomes. Criterion validity was explored for depression and PTSS by comparing mean scores on the HSCL-25 and HTQ among those who were identified by self and other local persons as having depression and PTS-like problems, respectively [29].

For depression symptoms, we used the 15-item HSCL-25 [17] depression subscale. Local adaptation included adding two items (“always stay alone” and “disappointed”), based on qualitative data suggesting these were important local depression-like symptoms. Respondents reported symptom frequency in the last month (0 [“none of the time”] to 3 [“almost always”]). An algorithm was applied to HSCL-25 results to determine study eligibility on the basis of moderate to severe depression. The HSCL-25 was also used to measure the depression severity outcome: scores on the depression subscale were calculated as the average symptom score across the 17 items and therefore ranged from 0 to 3. Internal consistency (α), measured from baseline trial assessments (*n* = 347), and test-retest/inter-rater reliability (*r*), measured locally prior to the start of the trial during the validation study, were acceptable (α = 0.79, *r* = 0.84) [29].

PTSS were measured using the 30 symptom items of the HTQ [17]. Local adaptation included adding specific Burmese language phrases from qualitative data to statements in the instrument, for example, “face is sweating, heart beats quickly” was added to the standard statement “sudden emotional or physical reaction when reminded of the most hurtful or traumatic event,” in order to increase clarity of the statement after translation. A total of ten items in the HTQ were adapted with specific local language. Response options were the same as in the HSCL-25. An algorithm was applied to HTQ results to determine eligibility on the basis of moderate to severe PTSS. The HTQ was also used to measure the PTSS severity outcome: scores for PTSS were calculated as the average symptom score across the 30 items and therefore ranged from 0 to 3. Internal consistency (α), measured from baseline trial assessments (*n* = 347), and test-retest/inter-rater reliability (*r*), measured locally prior to the start of the trial during the validation study, were acceptable (α = 0.84, *r* = 0.78) [29].

#### Secondary outcome measures

Functional impairment was measured using locally developed, sex-specific scales following methods described elsewhere [32]. Items were tasks that respondents in the prior qualitative study reported doing regularly to care for themselves, their families, or their communities (e.g., working for income, going to the market). The scales contained 16 and 23 tasks for men and women, respectively. Respondents reported current difficulty compared to others of the same sex and similar age (from 0 [“no difficulty”] to 4 [“often cannot do”]). Scores were calculated as the average task score across the 16- and 23-item scales and therefore ranged from 0 to 4. Internal consistency (α), measured from baseline trial assessments (*n* = 347), and test-retest/inter-rater reliability (*r*), measured locally prior to the start of the trial during the validation study, were acceptable (men: α = 0.90, *r* = 0.89; women: α = 0.92, *r* = 0.86) [29].

For anxiety symptoms, we used the ten-item HSCL-25 anxiety subscale [17]. Local adaptation included removing one item (“headaches”) and adding two items (“feel stressed” and “distrust, feel suspicious”) based on the prior qualitative and instrument validation studies. Respondent instructions and response categories were the same as for the HSCL-25 depression subscale. Scores were calculated as the average symptom score across the 11-item scale and therefore ranged from 0 to 4. Internal consistency (α), measured from baseline trial assessments (*n* = 347), and test-retest/inter-rater reliability (*r*), measured locally prior to the start of the trial during the validation study, were acceptable (α = 0.81, *r* = 0.86) [29].

For aggression, the 12-item Aggression Questionnaire [33] was adapted for local use. Respondents rated frequency in general of aggressive behaviors from 0 (“none of the time”) to 4 (“almost all of the time”). Scores were calculated as the averages score for each behavior across the 12-item scale and therefore ranged from 0 to 4. Internal consistency (α), measured from baseline trial assessments (*n* = 347), and test-retest/inter-rater reliability (*r*), measured locally prior to the start of the trial during the validation study, were acceptable (α = 0.73, *r* = 0.86) [29].

For alcohol use, we used the Alcohol Use Disorders Identification Test (AUDIT) [34]. Respondents reported frequency and amount of alcohol consumed, referencing photographs of local alcohols (local beers, rice whiskeys, etc.). No adaptations were made to these items based on qualitative data. Total scores were calculated as the sum total across the ten-item scale and ranged from 0 to 40. Internal consistency (α), measured from baseline trial assessments (*n* = 347), and test-retest/inter-rater reliability (*r*), measured locally prior to the start of the trial during the validation study, were acceptable (α = 0.80, *r* = 0.86) [29].

#### Other measures

We also recorded participant sex, age, marital status, ethnicity, education, current employment, number of people living in household, number and types of traumatic events either witnessed or experienced, current problems (six items: food insecurity, negative workplace experiences, fear of police harassment, fear of detention, financial difficulties, and social relationship problems), years living in Mae Sot, and number of close friends.

### Sample Size

We estimated needing 150 participants in each arm using the test for paired means, based on a moderate effect size (0.50), 80% power, a two-tailed 5% significance level, a design effect of 1.5, and an expected dropout rate of up to 50% (due to frequent cross-border movement). We used 1.5 for the design effect as indicating a moderate effect, given that we had no similar studies to compare to.

### Screening, Baseline Assessments, and Randomization

Rolling admissions were from August 8, 2011, to October 2012, ending when the sample size was achieved. The first baseline assessment was completed in August 2011, and the last follow-up assessment for enrolled clients was in November 2012. Counselors, partner staff, and locally knowledgeable persons referred persons for screening. Counselors visited referrals to obtain oral informed consent and to conduct the screening interview using the HTQ symptom scale and the HSCL-25 depression subscale. Inclusion criteria for moderate to severe depression and/or PTSS were defined using existing scoring algorithms for the HSCL-25 depression subscale and HTQ symptom scale based on DSM IV criteria [17]. We modified the original PTSS algorithm to be less stringent while still including persons with significant symptoms (Figure 1). For those found trial-eligible, oral informed consent for the trial was obtained, and the rest of the baseline assessment was completed. Given local sensitivity about signing documents and to better preserve confidentiality, counselors read an oral consent form both for the screening interview and trial participation. The counselor then recorded the person's decision and signed the consent form as a witness.

Each counselor then assigned participants the next available ID number from a block of 20 sequential participant ID numbers per counselor randomly allocated to intervention or wait-list control (WLC) status. The project site director generated these random numbers using STATA. Counselors opened a pre-sealed envelope (corresponding to the ID number) containing assignment to immediate treatment or wait-list. During their 3-4 month wait, controls received monthly calls from the project coordinator to check their safety and contact information. Safety checks included suicidal risk and implementation of a safety protocol when indicated [9]. After treatment or the wait-list period, interviewers not otherwise involved in the study conducted post-intervention assessments while masked to treatment/control status and baseline scores.

### Analysis

Treatment impact was derived using longitudinal modeling of within-person change in mean scores on the depression, PTSS, functional impairment, anxiety, aggression, and alcohol use scales from baseline to follow-up. Analysis of alcohol use was limited to persons with AUDIT scores of eight or above, the standard cutoff for harmful alcohol use [34], because only this group received the specific CETA component aimed at reducing alcohol use. Persons with AUDIT scores less than eight did not receive treatment for alcohol use (and therefore are not included in the analysis) because these scores are more likely to represent socially and personally acceptable alcohol use that is not problematic.

Statistical analyses used STATA 12.0. All outcomes were treated as continuous. A random effects model, with robust estimate of variance, was used to estimate treatment effects including time point (0 = baseline; 1 = follow-up) and counselor ID number as random effects to account for within-person correlation across time and between-person correlation by counselor. Missing data, including follow-up scores for those lost to follow-up, were imputed using STATA's chained equations command for multiple imputation, which pools data according to Rubin's rules [35],[36]. Briefly, missing at random (MAR) was assumed for the imputation model, using 11 imputations. First, any missing data on demographic variables were imputed based on all other demographic variables, the counselor ID number, and treatment status. Baseline and follow-up scores on all items missing data were then imputed using all of the variables in the dataset. Treatment and control groups were imputed separately. Average scores for all outcome variables were then calculated in the multiple imputation framework using all 11 imputed datasets. We did not do any data transformations. All final outcome models were run across the 11 imputed datasets.

Statistical significance was set at *p*<0.05, two-tailed, expressed as a 95% confidence interval. Cohen's *d* effect sizes, which reflect the size of effect over and above the change that occurred in the WLC condition, were calculated by dividing the difference in average change from baseline to follow-up for each outcome between treatment and control by the outcome's pooled standard deviation at baseline. Effect sizes are equivalent to a *Z*-score of a standard normal distribution; effect sizes of 0.2 are generally considered small, 0.5 medium, and 0.8 or above large [37]. All analyses used the full intent-to-treat (ITT) sample.

#### Adjusted models

In the adjusted models, all final outcome models except the alcohol use model were adjusted (the alcohol use model was left unadjusted because of small sample size) to account for possible residual confounding. Variables for which there was a significant difference between study arms at baseline, variables that were associated with the outcome (*p*<0.10), and variables known in the literature to be confounders of the relationship between treatment and outcome were included in the models. *t*-Tests for continuous variables and χ^2^ tests for categorical variables were used to test baseline differences. Univariate logistic regression was used to explore the association of variables with outcomes. In addition, the variable NGO affiliation (0 = AAPP; 1 = SAW; 2 = MTC) was added to the models to account for possible similarities of clients and counselors who were affiliated with each of the three NGOs involved in the study. To explore whether NGO affiliation should be treated as a random or fixed effect, a Hausman test [38] was utilized, with significance set at *p*<0.05.

Baseline anxiety was identified as being the only measured variable likely to be different between the two groups at baseline and was included in all adjusted models. All adjusted models included adjustment for sex, marital status, age, and education, based on the literature. Two outcome models, depression and impaired function, were adjusted for additional variables associated (*p*≤0.10) with the outcome. The depression model was adjusted for ethnicity, and the impaired function model was adjusted for types of traumatic experiences, time in Mae Sot, score on current problems index, and time between baseline and follow-up. NGO affiliation was also included in all models, as the Hausman test indicated it should be treated as a fixed effect rather than a random effect.

#### Sensitivity analysis

To test the robustness of the results with the assumption of MAR and using multiple imputation to impute follow-up scores, a sensitivity analysis was performed using a procedure described by White et al. [39]. This process tests whether the assumption of MAR (i.e., that people who are lost to follow-up are similar to people retained in the trial) in the main analysis is valid by examining whether the results are the same if the individuals lost to follow-up have systematically worse outcomes than those retained in the study. To perform this analysis, we added two standard deviation units to the imputed outcome scores for those lost to follow-up across all 11 imputed datasets for the primary outcomes of depression and PTS, and for the secondary outcome of functional impairment. We then reran the final models to determine whether the effects of the intervention were maintained.

#### Exploratory analyses

Additional exploratory analyses were conducted on trial outcomes (except alcohol use) by sex and for clients presenting with severe symptoms (defined as those scoring in the top 25th percentile on the HSCL-25 depression subscale or the HTQ symptom scale at baseline). To explore differences in treatment effects by sex, it was first determined whether there was a significant difference in effect between men and women, by testing a three-way interaction between time point (baseline and follow-up), treatment status (treatment or control), and sex. If the three-way interaction term was significant, then differences in effect sizes were explored. The analysis involving those clients with more severe symptoms was done to explore whether treatment effects were maintained among those with more clinically significant problems. These analyses are presented as exploratory only, since the study was not powered to do subgroup analyses.

## Results

### Sample Analyzed

**Figure 2:**
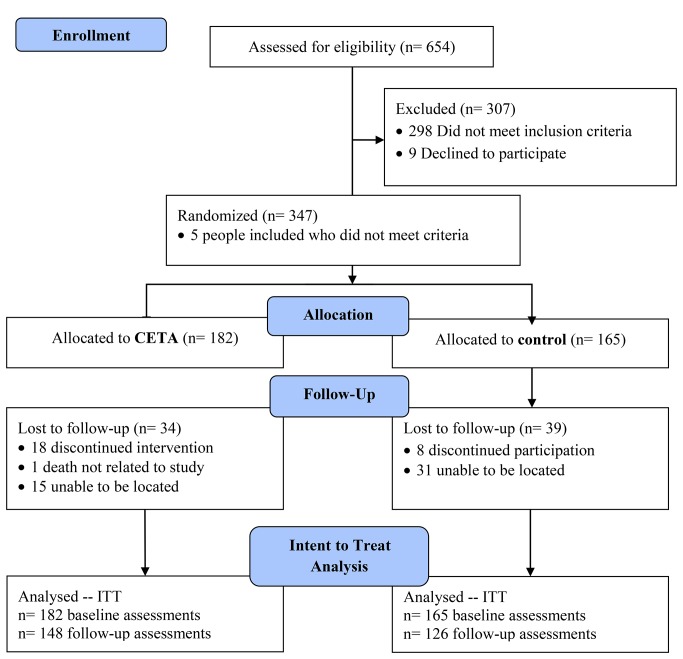
Flowchart of participants.

The ITT sample included 347 participants with baseline assessments and 274 (79%) with follow-up assessments (Figure 2). Five participants did not meet either the depression or PTS criterion (recruited in error) but were still included to remain consistent with the principles of an ITT analysis. Of 34 lost to follow-up in the intervention group, 18 withdrew from the intervention because of lack of time, a return to Myanmar, or a change in circumstances; one died; and 15 could not be located. Of 39 controls lost to follow-up, eight withdrew (five left Mae Sot or no longer had time), and 31 could not be located. Those lost to follow-up had higher baseline alcohol use, reported more current problems, were more likely to be of an ethnicity other than Burman, and were more likely to be recruited by the SAW and MTC organizations than by the AAPP organization. Full comparison of those lost to follow-up and those retained in the trial can be found in Tables S1 and S2. The rate of missing data for those who remained was 0.3%.

### Baseline Characteristics

Of 347 participants, 217 (63%) were female, average age was 35.6 y, 193 (56%) reported a high school degree or higher, 170 (49%) were currently married, and 220 (63%) were ethnically Burman (Table 2), the majority ethnic group in Myanmar. The high proportion of Burmans was because most participants were former political prisoners or their family members living in urban Mae Sot. These former political prisoners were typically involved in political demonstrations originating in central Myanmar cities where the majority of the population is Burman. We did not sample the refugee camps, which contain a larger proportion of ethnic minorities (i.e., non-Burmans) displaced by the many years of conflict between the central government and these groups. Participants had lived in Mae Sot for a mean of 5.6 y, and 206 (59%) reported current employment.

**Table 2:** Baseline characteristics (*n* = 347).

Characteristic	Subcategory	CETA Arm	Control Arm
**Sample size**		182 (52.5)	165 (47.5)
**Sex**	Male	71 (39.0)	59 (35.8)
	Female	111 (61.0)	106 (64·2)
**Marital status**	Not married^a^	82 (45.0)	89 (54.0)
	Married	98 (53.9)	72 (43.6)
	Missing	2 (1.1)	4 (2.4)
**Ethnicity**	Burman	121 (66.5)	99 (60.0)
	Other^b^	49 (26.9)	52 (30.5)
	Missing	12 (6.5)	14 (8.5)
**Education**	None	11 (6.0)	16 (9.7)
	Primary/middle school	72 (39.6)	55 (33.3)
	High school	50 (27.5)	49 (29.7)
	More than high school	49 (26.9)	45 (27.3)
**Current employment**	Unemployed	109 (59.9)	97 (58.8)
	Employed	72 (39.6)	64 (38.8)
	Missing	1 (0.5)	4 (2.4)
**Number of people in household**	1–10	115 (64.9)	107 (64.9)
	10–20	46 (25.3)	34 (20.6)
	>20	21 (11.5)	24 (14.6)
**Age, mean (SD), range**		36.5 (12.6), 18–85	34.3 (11.4), 18–65
**Number of traumatic events either witnessed or experienced, mean (SD), range**		12.1 (7.9), 1–24	11.9 (8.2), 1–24
**Number of current problems, mean (SD), range** c		3.6 (1.6), 0–6	3.7 (1.4), 0–6
**Years in Mae Sot, mean (SD), range**		5.4 (5.0), 0–35	5.7 (4.5), 0–25
**Number of close friends, mean (SD), range**		1.6 (1.7), 0–10	1·9 (2.9), 0–30
**Time on study (in months), mean (SD), range**		3·0 (1.2), 1.1–9.8	3.0 (1.2), 2.0–9.8

Data are *n* (percent) unless otherwise indicated. Student's *t* tests and χ^2^ tests found no significant differences between treatment and control groups (*p*<0.05).

a “Not married” category included *n* = 109 single, *n* = 24 widowed, and *n* = 38 divorced (in both treatment and control groups).

b “Other” category includes *n* = 68 Karen, *n* = 2 Kayah, *n* = 2 Kachin, *n* = 16 Mon, *n* = 5 Chin, *n* = 4 Rakhine, and *n* = 4 Shan (in both treatment and control groups).

c Current problems index included six items: food insecurity, negative workplace experiences, fear of police harassment, fear of detention, financial difficulties, and social relationship problems.

SD, standard deviation.

#### Suicidal risk

Twenty participants reported having thought about suicide “most of the time” (*n* = 14) or “almost all of the time” (*n* = 6), on the baseline assessment form. All were assessed for severity of risk and whether the safety protocol needed to be enacted. Eleven indicated no significant current risk (not thinking about killing themselves, no plan, no way to carry out plan, and never having tried). Seven others indicated that they thought about killing themselves, but did not have a plan or a way to carry out the plan, and had never tried committing suicide. No further action was taken for these 18 individuals. Two people gave an indication of greater risk and were subsequently visited by the clinical supervisor: one was determined to not have current risk, and no further action was taken; the other person was continually monitored by the project staff. No participants committed suicide during the study.

### Primary Outcomes

Both study arms experienced significant improvements in both primary outcomes (Tables 3 and 4). Unadjusted results indicated that the mean difference in improvement from pre- to post-intervention for depression symptom scores between treatment and control groups was −0.49 (95% CI: −0.59, −0.40), with a corresponding effect size of *d* = 1.16. The mean difference in improvement from pre- to post-intervention for PTSS scores was −0.43 (95% CI: −0.51, −0.35), with *d* = 1.19. Using adjusted models—adjusted for baseline anxiety, age, sex, NGO affiliation, and education in all models, and for covariates significantly associated with each specific outcome—the results were similar (Table 4).

**Table 3:** Average treatment effects (*n* = 347): unadjusted.

Outcome	CETA Arm		Control Arm		Net Effect		Effect Size Estimate^a^
	Mean Score	95% CI	Mean Score	95% CI	Mean Difference between Scores	95% CI	
**Depression (range: 0–3)**							
Baseline	1.33	1.21,1.44	1.27	1.16, 1.37			
Follow-up	0.31	0.24, 0.38	0.74	0.64, 0.85			
Pre-post change	−1.02	−1.12, −0.91	−0.52	−0.63, −0.42	−0.49**	−0.59, −0.40	1.16
**PTS (range: 0–3)**							
Baseline	1.06	0.97, 1.15	0.99	0.88, 1.10			
Follow-up	0.26	0.18, 0.33	0.62	0.51, 0.72			
Pre-post change	−0.80	−0.88, −0.72	−0.38	−0.47, −0.28	−0.43**	−0.51, −0.35	1.19
**Anxiety (range: 0–4)**							
Baseline	1.17	0.99, 1.35	1.03	0.86, 1.19			
Follow-up	0.28	0.18, 0.37	0.61	0.46, 0.77			
Pre-post change	−0.90	−1.05, −0.74	−0.42	−0.59, −0.24	−0.48**	−0.61, −0.34	0.79
**Functional impairment (range: 0–4)**							
Baseline	0.96	0.76, 1.17	0.88	0.68, 1.08			
Follow-up	0.33	0.24, 0.42	0.66	0.52, 0.80			
Pre-post change	−0.64	−0.83, −0.44	−0.22	−0.40, −0.03	−0.42**	−0.58, −0.27	0.60
**Aggression (range: 0–4)**							
Baseline	0.64	0.56, 0.72	0.65	0.53, 0.76			
Follow-up	0.17	0.12, 0.23	0.42	0.33, 0.50			
Pre-post change	−0.47	−0.53, −0.41	−0.23	−0.33, −0.13	−0.24**	−0.34, −0.15	0.58

“Pre-post change” is the change from pre-intervention to post-intervention.

a Measured using Cohen's *d* statistic and pooled baseline variances.

**p<0.001.

**Table 4:** Average treatment effects (*n* = 347): adjusted for outcome specific covariates.

Outcome	CETA Arm		Control Arm		Net Effect		Effect Size Estimate^a^
	Mean Score	95% CI	Mean Score	95% CI	Mean Difference between Scores	95% CI	
**Depression (range: 0–3)**							
Baseline	1.33	1.25, 1.42	1.29	1.21, 1.37			
Follow-up	0.32	0.25, 0.38	0.77	0.66, 0.87			
Pre-post change	−1.02	−1.12, −0.91	−0.52	−0.64, −0.41	−0.49**	−0.59, −0.40	1.16
**PTS (range: 0–3)**							
Baseline	1.06	1.00, 1.12	1.03	0.92, 1.10			
Follow-up	0.26	0.19, 0.32	0.64	0.54, 0.74			
Pre-post change	−0.80	−0.88, −0.72	−0.38	−0.47, −0.28	−0.43**	−0.51, −0.35	1.19
**Anxiety (range: 0–4)**							
Baseline	1.19	1.02, 1.35	1.03	0.88, 1.19			
Follow-up	0.29	0.21, 0.37	0.61	0.47, 0.76			
Pre-post change	−0.90	−1.05, −0.74	−0.42	−0.59, −0.25	−0.48**	−0.61, −0.34	0.79
**Functional impairment (range: 0–4)**							
Baseline	0.96	0.79, 1.13	0.90	0.73, 1.06			
Follow-up	0.32	0.23, 0.41	0.70	0.56, 0.83			
Pre-post change	−0.64	−0.83, −0.45	−0.20	−0.39, −0.02	−0.44**	−0.59, −0.28	0.63
**Aggression (range: 0–4)**							
Baseline	0.66	0.61, 0.71	0.68	0.57, 0.78			
Follow-up	0.19	0.15, 0.23	0.45	0.37, 0.53			
Pre-post change	−0.47	−0.52, −0.41	−0.22	−0.32, −0.13	−0.24**	−0.34, −0.15	0.58

Model-estimated differences after adjusting for baseline anxiety, age, sex, NGO affiliation, and education in all models and for covariates significantly associated with each specific outcome. All models include multiple imputation by chained equations for missing data and for missing outcomes due to loss to follow-up. Robust standard error estimators are used to account for clustering by counselor. “Pre-post change” is the change from pre-intervention to post-intervention.

a Measured using Cohen's *d* statistic and pooled baseline variances.

**p<0.001.

### Secondary Outcomes

Both study arms showed significant improvement on secondary outcome measures (Tables 3 and 4). In the unadjusted models, the mean difference in improvement from pre- to post-intervention scores between intervention and control groups was −0.42 (95% CI: −0.58, −0.27) for impaired function scores, −0.48 (CI: −0.61, −0.34) for anxiety symptom scores, and −0.24 (CI: −0.34, −0.15) for aggression behavior scores, with corresponding effect sizes of *d* = 0.60, *d* = 0.79, and *d* = 0.58, respectively. In the adjusted models, the only difference in estimates was for functional impairment, which showed an adjusted mean difference in improvement between intervention and control groups of −0.44 (95% CI: −0.59, −0.28), corresponding to an effect size of *d* = 0.63. Total alcohol use scores at baseline and follow-up for treatment and control groups were not normally distributed, so Table 5 displays the medians and interquartile ranges, as well as the average scores and treatment effects for this outcome. Participants in both treatment and control groups reported significantly less alcohol use at follow-up, with no significant difference between the groups (Table 5).

**Table 5:** Average treatment effects on alcohol use among alcohol users (*n* = 23).

Alcohol Use (Range: 0–4)	CETA Arm, *n* = 18				Control Arm, *n* = 15				Net Effect	
	Mean Score	95% CI	Median	IQR	Mean Score	95% CI	Median	IQR	Mean Difference between Scores	95% CI
Baseline	1.73	1.43, 2.03	1.55	1.0–2.4	1.69	1.38, 2.00	1.50	1.2–2.4		
Follow-up	0.48	0.24, 0.72	0.50	0–0.9	0.40	0.15, 0.65	1.0	0–0.5		
Pre-post change	−1.25	−1.54, −0.97			−1.29	−1.68, −0.89			−0.03	−0.44, 0.50

Reported baseline alcohol use score of eight or above on AUDIT; model weighted to account for loss to follow-up, and robust standard error estimators used to account for clustering by counselor. “Pre-post change” is the change from pre-intervention to post-intervention.

IQR, interquartile range.

### Sensitivity Analysis

Increasing depression, PTS, and impaired function outcome scores for those lost to follow-up by two standard deviations did not change the interpretation of results. Treatment effects were maintained for depression (mean difference at follow-up between intervention and control = −0.54, *p*<0.001), PTS (mean difference at follow-up between intervention and control = −0.46, *p*<0.001), and impaired function (mean difference at follow-up between intervention and control = −0.48, *p*<0.001). These results suggest that the assumption of MAR for those lost to follow-up is valid.

### Exploratory Analyses

Although the study was not powered to explore sex differences, the results suggest that there are no statistically significant differences in effect for men and women on any of the outcome measures.

The results from the analysis of the more severely symptomatic sample are likewise suggestive only, as the study was not powered to explore these differences. Results suggest that intervention effects for the more severely affected sample (*n* = 112 participants) were not qualitatively different from those of the whole sample for depression (*d* = 1.44), PTS (*d* = 1.61), functional impairment (*d* = 0.80), anxiety (*d* = 1.05), or aggression (*d* = 0.60) (Table 6).

**Table 6:** Adjusted treatment effects stratified by affected sub-population (severe: *n* = 112; moderate: *n* = 235).

Outcome	CETA Arm		Control Arm		Combined		Effect Size Estimate^a^
	Difference in Mean Score between Baseline and Follow-Up	95% CI	Difference in Mean Score between Baseline and Follow-Up	95% CI	Mean Difference in Change in Score between CETA and Control Arms	95% CI	
**Depression**							
Severe	−1.38**	−1.49, −1.26	−0.88**	−1.08, −0.67	−0.50**	−0.66, −0.33	1.44
Moderate	−0.82**	−0.92, −0.71	−0.38**	−0.49, −0.28	−0.44**	−0.56, −0.32	1.66
**PTS**							
Severe	−1.12**	−1.21, −1.02	−0.61**	−0.79, −0.43	−0.51**	−0.69, −0.32	1.61
Moderate	−0.63**	−0.72, −0.54	−0.29**	−0.39, −0.18	−0.34**	−0.46, −0.23	1.39
**Anxiety**							
Severe	−1.24**	−1.47, −1.00	−0.52**	−0.80, −0.23	−0.72**	−0.97, −0.47	1.05
Moderate	−0.71**	−0.85, −0.56	−0.38**	−0.54, −0.21	−0.33**	−0.49, −0.17	0.69
**Functional impairment**							
Severe	−0.81**	−1.10, −0.53	−0.19	−0.48, 0.09	−0.62**	−0.93, −0.31	0.80
Moderate	−0.54**	−0.72, −0.35	−0.22*	−0.45, −0.01	−0.31**	−0.48, −0.14	0.51
**Aggression**							
Severe	−0.60**	−0.71, −0.49	−0.33**	−0.52, −0.15	−0.27*	−0.44, −0.09	0.60
Moderate	−0.40**	−0.47, −0.32	−0.19**	−0.29, −0.09	−0.21**	−0.32, −0.10	0.56

Model-estimated differences after adjusting for baseline anxiety, age, sex, NGO affiliation, and education in all models and for covariates significantly associated with each specific outcome. All models include multiple imputation by chained equations for missing data and for missing outcomes due to loss to follow-up. Robust standard error estimators are used to account for clustering by counselor.

a Measured using Cohen's *d* statistic and pooled baseline variances.

*p<0.05;

**p<0.001.

### CETA Implementation

During the training, three of the four pre-identified locally based supervisors showed adequate uptake of CETA skill implementation as evidenced by role-play demonstration of CETA elements and, during the second half of the training, by their ability to coach counselors in role plays and in element selection and sequencing based on client vignettes. All three also demonstrated adequate supervisory skills, per their answers and ideas during additional supervision-specific training and by role-play demonstration of supervisory techniques (e.g., coaching in role plays, prompting objective reporting). One supervisor was not able to demonstrate supervisory skills and also was not fluent in English, rendering the Internet call supervision with trainers difficult. For these reasons, this supervisor served as a counselor rather than a supervisor. The three supervisors determined that two counselors needed a second pilot case before they had the skill to treat RCT participants.

For participants who completed CETA (*n* = 143; 79% of CETA participants), the average number of weekly sessions was 9.7 (range: 7–13). All participants received the CETA components engagement, psychoeducation, cognitive coping/restructuring, imaginal gradual exposure, and safety, and a closing session. The most commonly added components were behavioral activation (*n* = 29), SBI for substance use (*n* = 11), and anxiety management (*n* = 9).

## Discussion

### Summary of Results

This RCT evaluated the impact of a transdiagnostic treatment approach (CETA) on comorbid mental health problems and functioning among Burmese survivors of trauma. Treatment was provided by local counselors within the community because of lack of professional mental health services and the frequent reluctance of clients to attend clinics for fear of apprehension by Thai authorities. Compared to a WLC comparison condition, CETA was effective in reducing symptoms of depression, PTS, and anxiety. Effects were moderate for functional impairment and aggressive behaviors and negligible for alcohol use. Baseline symptom severity did not modify effects.

### Generalizability of Results

Our effect sizes were large to moderate, but this should be considered in comparison to (1) other transdiagnostic interventions and (2) other EBTs in low-resource settings, taking into account the type of comparison group and provider type. CETA effect sizes were similar to (depression and social functioning) or slightly smaller than (anxiety) those from a small RCT of an adult-focused transdiagnostic treatment in the US that also used a WLC [22] (0.97 for depression, 1.20 for social functioning, 0.54 for anxiety). A potentially important distinction is that in the Farchione et al. [22] trial, providers were mental health professionals under the supervision of PhD-level supervisors.

We examined results from other RCTs of EBTs in other low-resource settings that used a WLC condition—studies in which the providers were non-professionals (as in our study) and the treatments were structured EBTs. A study of IPT in Uganda showed a calculated effect size of 1.87 for depression [40]. Narrative Exposure Therapy showed moderate effects compared to a “monitoring only” group (similar to our WLC), with a calculated effect size of 0.53 at follow-up [41].

The magnitude of the average change in scores suggests clinical relevance based on local validity data and existing literature on the assessment measures [17]. Baseline scores for the treatment group were moderate to high in PTS and depression, and dropped to low levels (0.26 for PTS, 0.31 for depression). The control group also showed baseline scores of moderate to high PTS and depression but with a more moderate drop in scores at follow-up (0.62 for PTS, 0.74 for depression).

### Cultural Competence and Acceptability

Prior to the RCT we conducted preliminary research to adapt both instruments and intervention to the culture and situation of the participants [16]. All counselors and supervisors were Burmese refugees, sessions were conducted in native languages in familiar settings, and the intervention was tailored to the specific needs of the clients. While CBT skills are typically viewed as “Western” in origin, we note that some skills are based in Eastern traditions. For example, “relaxation” (anxiety management) is consistent with Burmese forms of meditation and was already commonly used by clients in meditation to decrease stress and improve well-being. This practice was encouraged by counselors in session. Similarly, after learning about the connection between thoughts and feelings, and more specifically about how to change negative, unhelpful thoughts to more positive, realistic thoughts (i.e., cognitive restructuring), one supervisor commented that this approach was very consistent with Buddhism because Buddhist teachings emphasize “balance” and “harmony,” not extreme thoughts or behaviors. Likewise, for another CBT skill, behavioral activation, in which clients increase pleasurable activities and reengage with their environment, counselors emphasized helping others or altruism, strengthening relationships with family members, and building connections with community members and organizations as ways for clients to feel better and participate in traditional activities. The most critical component of treatment, which all clients received, was imaginal gradual exposure for traumatic memories, in which counselors helped clients habituate to memories of trauma by helping clients tell their stories of trauma. During the training, many counselors and supervisors expressed concern about this component because clients would need to reveal personal details about themselves and their lives, which is in opposition to traditional Burmese practices. Contrary to expectations, clients were able to speak honestly about their traumatic experiences and expressed relief after sharing their difficult memories [42]. The results of this trial add to the literature suggesting that Western EBT can be appropriate and effective in other cultures when appropriately adapted.

### Contribution to the Literature

To our knowledge, this trial is the only published study to date testing delivery of a transdiagnostic intervention using a task-sharing approach in a low-resource setting. Previous studies of existing EBTs have demonstrated that non-professional workers can correctly and effectively provide highly structured interventions. In this study we found that these workers could go a step further and individualize treatment plans on the basis of presentation, in order to directly address multiple problems: local supervisors and counselors—with little prior mental health experience—made most of the decisions about which elements to deliver with specific clients, sequencing of elements to maximize client improvement, and dosing (e.g., how many sessions of each element were needed based on client understanding and symptom improvement). Counselors were able to implement CETA with fidelity, per local supervisor report. Our results suggest that this approach was effective compared to WLCs and that it was acceptable, as loss to follow-up was lower in the CETA arm (*n* = 34) than in the control arm (*n* = 39).

### Limitations

Loss to follow-up was higher than previous counseling intervention trials in low-resource settings [4],[6],[43]. Higher losses are likely due to the high population mobility characteristic of migrant and displaced populations, rather than to problems with the intervention, since losses were greater among WLCs (23.6%) than in the intervention group (18.7%). It is quite likely that the missing data have affected our outcome estimates, including the effect sizes, although the size and direction of the change is not known.

Assessors, but not participants, were masked, creating possible biases based in participant expectations. Because of population mobility, follow-up assessments were conducted at one time point only, close to CETA completion. Whether effects are maintained over time is unknown.

Mean baseline scores were low on all scales, suggesting that the overall sample was mildly to moderately affected by mental health problems. This may be a measurement artifact related to how the instruments perform in this population or the result of recruitment based on algorithms rather than scores. Although the sub-analysis examining those with higher scores found similar effects, we cannot claim to have studied a highly affected sample.

We chose to compare CETA to WLCs rather than an active control group. We understand that active controls are preferable when there is an existing standard of treatment that is known to be effective. We could find no prior research on the effectiveness of any mental health intervention among this population, nor was there a mental health or psychosocial intervention in common use. We did consider having an active control group consisting of clients meeting weekly with counselors who did not have CETA training, to account for the effect of weekly meetings. We rejected this as being unstandardized in terms of approach and content. In other words, we would not be able to say what it was that we were comparing the intervention to. Standardization would have required developing specific materials and training and supervising our workers in both the intervention and monitoring. Given the lack of literature supporting nonspecific interventions, it would have been difficult to justify this to our NGO partners.

Another option would have been to implement a specific EBT as an active control. We rejected this option because it would have produced a study of comparative effectiveness. Using such a design, it would not have been possible to demonstrate whether either intervention was effective: both interventions could show improvement from pre- to post-assessment because of simple regression to the mean. Only by having a true control group and subtracting the change from that group did we feel that the basic question could be answered of whether the intervention was effective, ineffective, or even harmful. We do not regard this as just a theoretical concern. In previous studies where we used control groups, the controls showed significant improvement [5],[6],[40], likely due in large part to regression to the mean. In unstable situations such as this one, where changes in the environment could affect our study outcomes, the risk of misinterpretation without a true control group is enhanced. The resulting use of a WLC group of individuals who did not receive weekly sessions or other treatment does create a problem in that the effect sizes we have reported refer to all aspects of the provider–client interaction and not just CETA. We do not know how much of the impact of the intervention was due to regular attention and support from a counselor versus treatment content (i.e., CETA). We are also unable to identify which aspects of CETA were most responsible for symptom improvement. To identify the most critical elements, future studies need to include either dismantling approaches or a substantially larger sample size to allow for sub-analyses of outcomes for different participant profiles and CETA elements received.

Finally, the trial employed levels of outside (i.e., US-based) monitoring and supervision that may not be feasible in an ongoing program. High levels were necessary to ensure that CETA was delivered with fidelity by newly trained providers, as this was a research study evaluating a new treatment. Compared to the authors' previous experiences implementing other EBTs in low-resource settings (e.g., IPT, trauma-focused CBT), the training and supervision of CETA was not more time-intensive. That is, the training involved the same number of days and practice, and supervision groups were conducted for approximately the same duration of time each week [6],[44]. However, these previous experiences were also research studies involving training new providers. At this time we do not know what level of monitoring and supervision would be required once providers and supervisors have completed their training and “apprenticeship.” For transdiagnostic treatments and other EBTs, supports needed for sustainability of delivering treatment with fidelity are unknown, limiting our ability to evaluate feasibility.

## Conclusions

A major challenge in global mental health is how to provide access to EBTs, particularly to persons with substantial comorbidity or residing outside urban areas. In most low-resource settings, an EBT model based on hospitals or referral to professional services cannot reach most people. In physical health this approach was long ago recognized as inadequate, resulting in its replacement by the primary health care model as the only feasible approach to widespread access to health care, particularly for poor and/or rural populations [45]. A few studies have taken up the challenge of providing EBTs at the primary or community level. Several have demonstrated effectiveness for multiple disorders when EBTs are provided by non-professional workers and therefore have presented viable community-based treatment options. We wondered whether non-professional providers could go further and adapt treatment to varying presentations, thereby providing an additional option for community-based treatment.

We undertook this study as a first test of a new approach to flexible treatment of varying and comorbid common mental health problems at the community level. We found that local counselors were able to apply this approach correctly and effectively. The results of this trial warrant the continued development and testing of transdiagnostic approaches among this population of Burmese displaced persons, as well as testing of these approaches in other low-resource situations where treatment access and comorbidity are important challenges. Future studies should include comparisons with existing treatments known to be locally effective, to begin to determine the specific role of the transdiagnostic approach to symptom and function improvement.

## Supporting Information

Table S1 Baseline characteristics (*n* = 347) comparing those retained in study and those lost to follow-up.
(DOCX)

Table S2 Average symptom scores at baseline comparing those retained in study and those lost to follow-up.
(DOC)

Text S1 Study protocol.
(PDF)

Text S2 CONSORT statement.
(DOC)

## Abbreviations

AAPP: Assistance Association for Political Prisoners–Burma
AMHR: Applied Mental Health Research Group
AUDIT: Alcohol Use Disorders Identification Test
CBT: cognitive behavioral therapy
CETA: Common Elements Treatment Approach
EBT: evidence-based treatment
HIC: high-income country
HSCL-25: Hopkins Symptom Checklist 25
HTQ: Harvard Trauma Questionnaire
IPT: Interpersonal Psychotherapy
ITT: intent-to-treat
MAR: missing at random
MTC: Mae Tao Clinic
NGO: nongovernmental organization
PTS: posttraumatic stress
PTSS: posttraumatic stress symptoms
RCT: randomized controlled trial
SAW: Social Action for Women
SBI: screening and brief intervention
WLC: wait-list control
