# Supplementary material for: A Transdiagnostic Community-Based Mental Health Treatment for Comorbid Disorders: Development and Outcomes of a Randomized Controlled Trial among Burmese Refugees in Thailand
Source: PLoS Med. 2014 Nov 11;11(11):e1001757. doi: 10.1371/journal.pmed.1001757 (PMC4227644; doi:10.1371/journal.pmed.1001757)
Supplement: Table S1 — Baseline characteristics (n = 347) comparing those retained in study and those lost to follow-up. (DOCX) [file pmed.1001757.s001.docx]

| **Table S1.**  *Baseline characteristics (N=347) comparing those retained in study and those lost to follow-up* | | | |
| --- | --- | --- | --- |
|  |  | **Retained** | **Lost to follow-up** |
|  |  | *N* (%) | *N* (%) |
|  | **Sample Size** | 274 (79.0) | 73 (21.0) |
| **Treatment Status** | Treatment | 148 (81.3) | 34 (18.7) |
|  | Control | 126 (76.4) | 39 (23.6) |
| **Sex** | Male | 100 (36.5) | 30 (41.1) |
|  | Female | 174 (63.5) | 43 (58.9) |
| **Marital Status** | Not married^b^ | 134 (48.9) | 37 (50.7) |
|  | Married | 134 (48.9) | 36 (49.3) |
|  | Missing | 6 (2.2) | 0 (0.0) |
| **Ethnicity** | Burman | 171 (62.4) | 49 (67.1) |
|  | Other^c^ | 87 (3.18) | 14 (19.2) |
|  | Missing | 16 (5.8) | 10 (13.7) |
| **Education** | None | 20 (7.3) | 7 (9.6) |
|  | Primary/middle school | 98 (35.8) | 29 (39.7) |
|  | High school | 78 (28.5) | 21 (28.8) |
|  | More than high school | 78 (28.5) | 16 (21.9) |
| **Current Employment** | Unemployed | 168 (61.3) | 38 (52.1) |
|  | Employed | 102 (37.2) | 34 (46.6) |
|  | Missing | 4 (1.5) | 1 (1.4) |
| **Number of people with whom you live** | 1-10 | 179 (65.3) | 43 (58.9) |
|  | 10-20 | 60 (21.9) | 20 (27.4) |
|  | >20 | 35 (12.8) | 10 (13.7) |
| **Age,** Mean (*S.D.*), *Range* |  | 35.6 (12.5)  *18-85* | 34.7 (10.7)  *18-65* |
| **Number of traumatic events either witnessed or experienced,** Mean (*S.D.*), *Range* |  | 11.9 (8.1)  *1-24* | 12.2 (7.9)  *1-24* |
| **Current Problems,** Mean (*S.D.*), *Range*^d^ |  | 3.5 (1.5)  *0-6* | 3.9 (1.4)  *1-6* |
| **Years in Mae Sot,** Mean (*S.D.***),** *Range* |  | 5.4 (4.5)  *0-35* | 5.9 (5.7)  *0.1-25* |
| **Number of close friends,** Mean (*S.D.*), *Range* |  | 1.8 (2.5)  *0-30* | 1.6 (1.6)  *0-7* |

^a^ Students t-tests and chi-squared tests found no significant differences between treatment and control groups (*p* < 0.05).

^b^ Not married category included *n =* 109 single; *n =* 24 widowed, *n =* 38 divorced (in both treatment and control groups)

^c^ Other category includes *n =* 68 Karen, *n =* 2 Kayah, *n =* 2 Kachin, *n =* 16 Mon, *n =* 5 Chin, *n =* 4 Rakhine, *n =* 4 Shan (in both treatment and control groups).

^d^ Current Problems index included 6 items: food insecurity, negative workplace experiences, fear of police harassment, fear of detention, financial difficulties, and social relationship problems
