# Supplementary material for: A Transdiagnostic Community-Based Mental Health Treatment for Comorbid Disorders: Development and Outcomes of a Randomized Controlled Trial among Burmese Refugees in Thailand
Source: PLoS Med. 2014 Nov 11;11(11):e1001757. doi: 10.1371/journal.pmed.1001757 (PMC4227644; doi:10.1371/journal.pmed.1001757)
Supplement: Table S2 — Average symptom scores at baseline comparing those retained in study and those lost to follow-up. (DOC) [file pmed.1001757.s002.doc]

| **Table S2.**  *Average symptom scores at baseline comparing those retained in study and those lost to follow-up* | | | |
| --- | --- | --- | --- |
|  | Retained  (*n = 274*) | Lost to follow-up  (*n = 73*) |  |
|  | *Mean (S.D)* | *Mean (S.D)* |  |
| **Depression** (*Range:* 0-3) | 1.31 (0.41) | 1.36 (0.43) |  |
| **PTS** (*Range:* 0-3) | 1.06 (0.37) | 1.04 (0.37) |  |
| **Anxiety** (*Range:* 0-4) | 1.11 (0.59) | 1.17 (0.66) |  |
| **Function** (*Range:* 0-4) | 0.93 (0.69) | 0.99 (0.73) |  |
| **Aggression** (*Range:* 0-4) | 0.66 (0.40) | 0.72 (0.46) |  |
| **Alcohol use** (*Range*: 0-10) | 1.83 (5.13) | 3.34 (6.70) |  |
