# Supplementary material for: A Transdiagnostic Community-Based Mental Health Treatment for Comorbid Disorders: Development and Outcomes of a Randomized Controlled Trial among Burmese Refugees in Thailand
Source: PLoS Med. 2014 Nov 11;11(11):e1001757. doi: 10.1371/journal.pmed.1001757 (PMC4227644; doi:10.1371/journal.pmed.1001757)
Supplement: Text S1 — Study protocol. (PDF) [file pmed.1001757.s003.pdf]

JHSPH Institutional Review Board
RESEARCH PLAN

PI: **Courtland Robinson**
Study Title: **Study of effectiveness of mental health interventions among torture survivors on the Thailand-Burma border**
IRB No.: 3601
PI Version Number/Date: **Version 4.0, June 13, 2011**

1. Aims/objectives/research question/hypotheses:

The aim of the study is to determine the effectiveness of a psychotherapeutic intervention – namely, Components-Based Intervention (CBI) – in reducing the severity of mental health symptoms experienced by torture and violence survivors displaced from Burma into Thailand. Specifically, the intervention seeks to measure reductions (if any) in symptoms of depression and trauma. These two problems were prominently identified in the qualitative research (IRB#3047) that preceded this intervention evaluation phase of the project. Depression symptoms will be measured using a modified, locally validated version of the Hopkins Symptoms Checklist (HSCL) and trauma exposure and symptoms will be measured using a modified, locally validated version of the Harvard Trauma Questionnaire (HTQ). The study seeks to compare the effect of the CBI intervention against no CBI intervention. We are not comparing the CBI intervention with other available services and treatments, which may include non-specific counseling services and/or inpatient care. While the study does not discourage any use of standard of care treatments, and will document any such services (including possible medication) in the client monitoring records, we do not make any hypotheses about what effects the presence or absence of other treatments would have on study results.

2. Background and rationale:

Little is known about the effectiveness of most mental health interventions among torture survivors. Interventions provided to survivors are usually based on limited evidence because few studies have specifically targeted survivors. This has led to controversy in the field of torture treatment between those who believe that the rationale for current treatments is appropriate and those who believe that torture survivors present a unique set of challenges that may render such treatments unsuitable. The few studies that have been done were mostly among torture survivors living in developed countries. They therefore face different challenges than those living in low resource environments and in the countries in which the torture occurred. In the case of the Southeast Asia—in particular the situation of Burmese populations experiencing torture and systematic violence—we know of no completed studies of treatments for torture survivors and therefore have no scientific evidence as to which interventions are acceptable to local people, which are feasible given local resources, and which are effective given the local culture and the other challenges in the lives of survivors. Therefore, in this study we are evaluating a psychotherapeutic intervention—namely, Components-Based Intervention (CBI)—to treat the mental health problems of torture survivors, drawing upon preliminary results from Johns Hopkins University (JHU) studies in Northern Iraq (IRB#2091), Southern Iraq (IRB #3034**),** and in consideration of the mental health problems described by displaced Burmese populations in previous JHU research on the Thailand-Burma border (IRB #3047, IRB#3348).

The study is part of an award by the USAID Victims of Torture Fund (USAID/VTF) to JHU to work with local and international organizations serving survivors of torture and systematic violence. For this study, the intent is to assist in the design, implementation, monitoring, and evaluation of programming to understand and address the psychosocial needs of Burmese displaced across the Thai/Burma border who are living in the area of Mae Sot, Tak Province in Thailand. Specifically this project involves collaboration with the Burma Border Project (BBP), the Mae Tao Clinic (MTC),

the Assistance Association for Political Prisoners (AAPP), and Social Action for Women (SAW), to help improve the quality and effectiveness of psychosocial and mental health programs.

The Components-Based Intervention includes psycho-education, relaxation, life-enhancing skills (problems solving and safety planning), family support, and cognitive processing of life problems and traumatic events. The CBI model is being tested in a JHU study in Southern Iraq and preliminary results suggest that it may be effective for the population of displaced Burmese in Thailand. The duration of sessions is one hour, the frequency of treatment is weekly, and the recommended number of sessions in the Thailand/Burma border site is 10.

In February-March 2011, US-based clinical psychologists trained 20 counselors and 4 supervisors in CBI techniques. The training curriculum involved 10 full days (8:00am – 4:00pm) of live training including didactic explanations, demonstration role-plays and small-group role-plays. CBI components covered in the training included:

1) Pre-Treatment (consent, intake/screening, and assessment)
2) Encouraging Participation (why mental health services are important)
3) Psychoeducation (information about problems and treatment process)
4) Behavioral Coping Skills: Relaxation (helping clients learn ways to cope with stress)
5) Behavioral Coping Skills: Behavioral Activation (helping clients to do activities they enjoy)
6) Cognitive Coping (counselors talk with clients about how thoughts, feelings and behavior are connected and how to think in different ways in order to change feelings and actions)
7) Gradual Exposure (for Trauma) (counselor engages client in a direct detailed discussion about traumatic events he/she has experienced and connected thoughts and feelings)
8) Live Exposure (for Fear-based Anxiety) (counselors talk with clients about slowly, step-by-step, facing a place/thing they are afraid of)
9) Cognitive Reprocessing (counselors challenge client's unhelpful and inaccurate thoughts)
10) Enhancing Safety (counselors ask clients specific questions about safety and suicidal ideation and work with client to develop and implement a safety plan)

Starting in June 2011, the counselors trained in CBI will begin to implement this intervention with the clients that come to see them as part of the ongoing services provided by AAPP, SAW, and MTC. A practicum round of the CBI intervention began shortly following the CBI training in order to be sure that counselors and supervisors are properly trained in the intervention. The research study will begin in June 2011 when approximately 400 people will be screened into the intervention (for a final sample of 300); approximately half of these would be randomly assigned to the "case" group, to begin the intervention immediately after screening while the other half would be assigned to the "wait-control" group to receive CBI after the "case" group has completed the CBI intervention.

The proposed study will be embedded within the ongoing MTC, SAW and AAPP programs which could include non-specific counseling services, social services and support and, in the case of Mae Tao clinic, medical assistance. That is, we are proposing to study the effectiveness of these intervention strategies in a subset of the clients the organizations serve. The organizations will continue to provide services, as space allows, to anyone who seeks their services regardless of whether they meet eligibility for our proposed study. However, those who meet our inclusion criteria will be invited to participate in the research component of the service program.

3. **Participants**:

Persons eligible for participation in this study will include the following: adults from Burma (18 and older) living in Mae Sot with significant mental health problems; who were affected by torture and systematic violence (that is either directly subjected to torture and imprisonment, or indirectly

through witnessing torture, or who are a close family member of a survivor) and who are not actively suicidal. Eligible persons will mainly be located with the assistance of our local organizational counterparts. Some may be former prisoners who were tortured in prison by the Burmese military regime. Others will be identified as they present to the local organizations seeking help for various problems, including mental or physical disorders. All those who are found to be eligible will be invited to join the trial.

Screening will be done using an instrument developed for use among this population (see attached document). For the baseline interview, Section 1 (Demographic Characteristics), Section 2 (Trauma Events), Section 3 (Depression Symptoms) and Section 4 (Trauma Symptoms) will serve as the screening tool to identify persons found to have experienced or witnessed torture or systematic violence and who exhibit significant symptoms of trauma or depression. This will comprise Part A: Screening Instrument of the baseline interview.

Those who are found to be eligible on exposure and symptoms criteria—and who are also eligible by being adults from Burma living in Mae Sot, Thailand—will be consented to continue the full intake/assessment interview and to join the trial. Part B of instrument comprises the intake/assessment portion, and includes Section 5 (Anxiety), Section 6 (Function Assessment), Section 7 (Current Problems), Section 8 (Aggressive Behavior) and Section 9 (Alcohol Use).

There may be some individuals who are deemed eligible and have consented to participate in the study but who, following the full intake interview, would be deemed excludable either because they are a danger to themselves or others, or who are psychotic or otherwise deemed not competent to give consent or participate in the interventions. While we believe the study intervention is not appropriate for these individuals, counselors will review the cases with their clinical supervisors and the JHU study team to ensure that every effort is made to provide these individuals with referral to appropriate care.

4. Study procedures:

1) General study design.

This is a two-arm randomized control trial design. Twenty local counselors and 4 clinical supervisors working with three local organizations in Mae Sot have been trained by JHU or other US-based clinical psychologists to provide the CBI intervention to all the survivors they treat. CBI is a ‘components-based’ intervention that includes psychoeducation, relaxation, life-enhancing skills (problems solving and safety planning), family support, and cognitive processing of life problems and traumatic events, in a specific way, and is based on the current standard of practice for trauma-affected clients. Approximately half of those found eligible for the trial will be randomly allocated to the case group to receive the intervention immediately and the other half will be asked to wait for approximately 3 to 4 months before being reassessed and then receiving the intervention being provided by the counselors. The wait-control group will form the second arm of the trial.

2) Study procedures, including their sequence and timing.

Recruitment: Beginning in May 2011, recruitment for the intervention effectiveness study will begin. Recruitment of persons for screening and enrollment will be done by contacting the local organizations and inviting program beneficiaries and community members to come to the counselors to be screened. Recruitment will be done by word-of-mouth and through routine program contacts with the community. Local partner organizations (BBP, AAPP, MTC and SAW) have developed a one-page handout and pamphlet in the local language (Burmese) that provides a brief description of the project and contact information for persons in the project whom they can contact for more information. These contact persons with the local partner

organizations will then collect names and contact information for those who express interest in possibly taking part in the study. The recruitment process will identify the availability of a new intervention for people who have experienced or witnessed torture or systematic violence and who feel they might benefit from counseling for depression or trauma symptoms. Potential participants will be told that the program is available for Burmese persons 18 years or older who will remain in the Mae Sot area for the duration of the intervention (10 weekly sessions). Persons expressing an interest in being contacted for a follow-up discussion will be invited to provide their contact information to the local program staff-members as mentioned above. We anticipate that the recruitment process will take approximately 4-8 weeks, to be carried out as counselors and clinical supervisors are completing a practicum period of training in the CBI method (March – June 2011). All contact information will be collected from the local partner organizations by the on-site JHU Project Director who will maintain the overall file of potential clients for the study. Local partner organizations will be asked to destroy their copies of the names and contact information as this will be maintained only by the Project Director on a secure file.

Training on Consent and Screening Procedures: We plan to begin the actual intervention study with CBI in June 2011 (corresponding to Month 5 in the table below). Prior to that, JHU study faculty will provide training to all counselors and clinical supervisors on how to carry out consent and screening procedures, including administering the study consent form and interpreting depression and trauma severity scores.

Screening: A screening process, and allocation to either an intervention (case) group or temporarily to a wait-control group, will continue until the practicum round of CBI training has been completed in late June or early July. If a sufficient number of clients for the intervention group are not identified by early July, screening and enrollment could continue beyond that time.

Service Provision: Beginning in late June or early July 2011, the intervention will begin with weekly sessions provided by the counselors to the client and with supervision provided in the form of weekly meetings between the counselor and clinical supervisor and in the form of weekly phone conversations between the clinical supervisors and the JHU trainers/clinical psychologists. Both intervention and control clients will be monitored using separate client monitoring forms. These forms will include information on the clients' well-being, any treatment or medication they are receiving, and whether or not the client's are in danger to themselves or others. Clients who are in danger of committing suicide will be discussed immediately with supervisors, or referred if supervisors are not immediately available. There is a Safety Plan (see attachment) in place for this project that includes a response team of the following: the counselor, the counselor's clinical supervisor, a medical doctor who is part of the project team, and the Field Project Coordinator. The Project Director will provide support for this response team at all times and will be in regular contact with the Project PI. A copy of the Safety Plan protocol is included in the IRB application.

Persons who are in the intervention group or the wait control group whose depressive symptoms worsen (but not to the point of ineligibility) would first be evaluated by the counselor in conjunction with the study's clinical team and with the CCT medical monitor. Based on this assessment, a decision could be made to continue CBI treatment alone or the client could be provided referral to standard of care treatment possibly including non-specific counseling. This treatment would be recorded in the client monitoring records.

We should note that some of the counselors who will deliver the CBI intervention are also qualified to provide other services (for example, some of the Mae Tao Clinic counselors providing the intervention also work at the Counseling Center on a part-time basis). We will ask for the period of the intervention, that the CBI counselors provide only CBI to their study clients. if referrals are made for other services (or clients independently seek other services including,

possibly, non specific counseling) these other services would be provided by someone other than the CBI counselor.

	Month											
	1	2	3	4	5	6	7	8	9	10	11	12
1. Training on CBI Method	XX	XX										
2. Practicum Round on CBI			XX	XX	XX							
3. Recruitment				XX	XX							
4. Screening and Baseline Interviews of Case and Wait-Control					XX	XX						
5. Intervention (Case Group)						XX	XX	XX	XX	XX		
6. Follow-up Interviews (Case and Wait-Control Groups)										XX		
7. Intervention for Wait-Control Group										XX	XX	XX
8. Study Wrap-up											XX	XX

Follow-up interviews. All follow-up interviews (including with the wait-control group) will be conducted at the end of the intervention for the case group, which would be no more than 3 to 4 months after the date of enrollment into the study.

Following completion of the trial, the data will be analyzed, and if the intervention is found to be effective, it will become part of the services provided by the counselors to torture and violence survivors.

3) *Number of study contacts or visits required of participants*

We expect at most 2 research contacts with each intervention participant for the purposes of the study - the initial screening and the post-intervention interview. Wait-control group individuals will also receive monthly contacts during their waiting time, totaling 3 to 4 contacts during the trial.

4) *Expected duration of the study*

We expect the study will last approximately 12 months, including the data analysis period. The two phases of the intervention—CBI for the case group and CBI for the wait-control group (if it is established as effective)—we expect to be concluded by the end of December 2011.

5) *A brief data analysis plan and description of the nature of the variables to be derived*

As noted previously, depression and trauma were the two mental health problems that were prominently identified in the qualitative research (IRB#3047) that preceded this intervention evaluation phase of the project. Depression symptoms will be measured using a modified, locally validated version of the Hopkins Symptoms Checklist (HSCL) and trauma exposure and symptoms will be measured using a modified, locally validated version of the Harvard Trauma Questionnaire (HTQ). The data generated from the validation study has been used to develop depression and trauma scale cutoff scores for the study population. The counselors will calculate the total score for these subscales during the initial screening interview, since scores on either of these scales above the cutoffs are part of the eligibility criteria (13 or above for

depression symptoms and 45 or above for trauma symptoms). Following randomization into a case group and a wait-control group, the counselors will provide the intervention to the case group. At the end of the intervention, the case group and the wait-control group will be re-interviewed using the same instrument employed in the screening interview. The mean change in the various subscales between pre and post intervention interviews will be calculated and subtracted from the mean change (calculated in the same way) for the wait-control group. The differences will be the amount of change on each scale due to the intervention.

In terms of statistical analysis, we will keep the data on study participants in the intervention group who get other standard of care treatment or participants in the wait-control group who need immediate treatment. All study participants who meet screening criteria for eligibility and enroll in the study will be included in the analysis on an intent to treat basis.

6) If human biospecimens (blood, urine, saliva, etc.) will be collected, provide details about collection, volume (ml) or number, use, storage, identification, and disposal.

Not Applicable

7) Describe how subjects will be screened for eligibility and assigned to study/intervention and comparison/control groups.

All adults who meet with one of the 20 trained counselors will be verbally consented and screened by the counselors for study eligibility. Counselors will first re-confirm that the person lives in Mae Sot, will be available for the duration of the study, and is 18 years or older. The screening interview will then be used to establish whether the individual meets our definition of exposure to torture or systematic violence (experienced or witnessed at least one trauma exposure event), and has significant depression and/or trauma symptoms (as noted previously, the scores are 13 or above for depression symptoms and 45 or above for trauma symptoms).

Exposure to torture and violence will be measured through a review of the trauma exposure questions on the assessment interview. There are 25 questions that ask about exposure to traumatic events. If the respondent indicates that they personally experienced or witnessed any of these for exposures, they will be regarded as having been exposed to torture for purposes of this study. If they are not eligible for our study, they can continue to receive counseling and support as per existing standards of care and service provided by the local participating organizations.

Measures of trauma or depression symptoms will be based on the responses to all trauma and depression questions in the screening interview. The counselors will calculate the total score for these questions (defined as the total trauma and depression subscales) while the interviewee is still present. If this meets or exceeds a cutoff score of 45 for trauma---reflecting likely clinically significant symptoms (based on results of a previous validity study of the instrument among the same population) and/or a cutoff score for depression of 13 or more ---reflecting likely clinical significant depression (again based on results of the previous validity study)---then the person will be invited to join the trial after having been read a consent form which explains the trial. If they agree they will be assigned either to receive treatment beginning soon or be told that they are on a wait-list and will need to wait several months before receiving treatment. If they are eligible but decline to participate in the trial, the counselor and clinical supervisor, in consultation with the JHU study team, will review treatment options on a case-by-case basis. Depending on the outcome of this assessment, these individuals could be offered to receive the study treatment (though not participate in the study) or they could be referred to other appropriate programs and services in the Mae Sot area.

While the study imposes no upper cut-off point for depression and trauma symptoms (and thus would not exclude persons with acute mental illness), there may be some individuals who are deemed eligible and have consented to participate in the study but who would be excludable either because they are a danger to themselves or others, or who are psychotic or otherwise deemed not competent to give consent or participate in the interventions. While we believe the study intervention is not appropriate for these individuals, counselors will review the cases with their clinical supervisors and the JHU study team to ensure that every effort is made to provide these individuals with referral to appropriate care.

In the case of individuals who evidence signs of active suicidal ideation, counselors and clinical supervisors have been trained on suicide assessment as part of their CBI training. In all cases involving potential danger of a client harming him/herself or others, counselors will follow the study Safety Plan to request assistance in assessing the need of follow-up care and services for that individual. On a case-by-case basis, the study team will work with the counselor and clinical supervisor, along with Dr. Htin Zaw and the study team's clinical experts, to determine whether to offer the individual the study treatment (if they are excludable from the study intervention), or the study intervention (if they are otherwise eligible and no longer deemed an active risk to harm themselves or others), or some other available and appropriate treatment.

Randomization process:
If the subject is deemed eligible and consents to join the trial, the subject will be randomly allocated to the case group to receive treatment immediately or to the wait-control group. The randomization process will be as follows:

1. Each counselor will receive a list of participant ID numbers in sequence (i.e. 1-15) which have been randomly allocated to either intervention or wait-control via a random number generator, with a 1 to 1 ratio of intervention to wait-list. There will be one list for each counselor. Counselors will not be aware how numbers have been allocated.

2. For each counselor, a paper indicating the treatment assignment (intervention or wait-list) will be folded and stapled directly to the back of consent forms that are pre-numbered with a client ID number.

3. Once the subject has consented to be in the study, the counselor will assign a number to the client in the order in which they are recruited. They will remove and unfold the paper attached to the form that corresponds to their ID number, and will inform the subject whether they will begin the intervention immediately or will be put on the wait list.

The study investigators will keep master lists for each counselor which indicate the allocated group status (intervention or wait-control) for each client ID number to ensure fidelity to the randomization model.

Persons in the wait-control group will be instructed to contact their counselor, either by phone or in person, if their symptoms worsen during the wait period. The Field Program Coordinator will work with the counselors to coordinate a process of check-in (either in person or by phone) with wait-control clients every month for assessment of symptom severity by the counselors. In cases where the symptoms are worse, this will be reported to the clinical supervisors and the JHU study team and a decision made about whether the participant should be referred for additional services. In severe cases where the person is a risk to themselves or others the project will follow the Safety Plan (explained above).

8) Explain and justify whether there will be blinding.

In this study, the follow-up interviews of the case group clients will be carried out by counselors different from those who provided the CBI treatment. Follow-up interviews will be randomly assigned to counselors who will be blind to the initial assessment scores of the interviewee and who would not have knowledge of a client's results during the CBI intervention. This will eliminate risk of bias on the part of counselors who might wish to improve the outcome scores of their client caseload and would promote rater independence between the pre- and post-intervention measures.

9) Explain and justify whether participants will not receive routine care or will have current therapy stopped

Standard care for Burmese displaced persons with mental health problems in Mae Sot could include treatment at Mae Tao Clinic's Counseling Center where treatment includes non-specific counseling and (frequently) medication. Cases involving active suicidal ideation or psychotic breakdown could be provided in-patient treatment at Mae Tao Clinic or referral to Mae Sot Hospital where psychiatric services are provided. Mae Sot Hospital is a facility primarily for the local Thai population and is not routinely used by the Burmese population for reasons of cost, insecurity (due to travel risks and possible contact with Thai authorities) and language incompatibilities. In the case of psychiatric services, this usage of Mae Sot Hospital by the Burmese migrant community has been limited generally to emergency cases involving, for example, a suicide attempt (or active suicidal ideation) or a severe psychotic episode, Mae Tao Clinic, on the other hand, is a site that Burmese migrants may be more likely to use for mental health services. MTC's Counseling Center provides on-site, non-specific counseling services and MTC also provides some inpatient care. Both the Counseling Center and the inpatient services tend to include some medication as part of treatment. Participation in the CBI intervention does not require that an individual discontinue other treatments or therapies nor would they be discouraged from using other services during the course of the study intervention. The study seeks to compare the effect of the CBI intervention against no CBI intervention. We are not comparing the CBI intervention with other available treatments. Thus, for individuals in the intervention group who may experience a worsening of symptoms or individuals in the wait-control group who need immediate services a decision to refer to standard of care treatment would be made on a case-by-case base following discussions by the treatment team—the counselor, clinical supervisor, and the clinical team (Dr Htin Zaw and the JHU clinical experts) Use of any such services, including medications (if any) would be discussed in the CBI counseling sessions and documented in the client monitoring records.

10) Explain and justify the use of a placebo or non-treatment group.

No placebos will be used. There will be a wait-control group who will not initially receive treatment. Instead they will wait until the intervention has been completed for the case group, a period of no more than 3 to 4 months. They will then be reassessed before beginning treatment. The reason for the initial non-treatment is that we do not have evidence of the effectiveness in this setting for the treatment included in the study.

Once wait-control group participants have waited the full intervention period (3 to 4 months), they will then receive the intervention from the counselor they saw at intake (unless it is established that the intervention is shown to be unhelpful or harmful). In addition, controls will be provided the study therapy if their symptoms become clinically significantly worse, or (in severe cases where the person is a risk to themselves or others) they may be referred to other sources of care (either Mae Tao Clinic or Mae Sot Hospital).

11) Provide a definition of treatment failure or participant removal criteria.

Clinical treatment failure is defined as an individual completing less than 6 sessions of the Components-Based Intervention. Available research suggests that this is a significant “dose” of therapy to see treatment effects. The success or failure of the intervention failure will not be assessed at the individual level but only at the group level. Failure at the group level for the intervention would consist of less than a 20% difference in change in mean symptom severity score between pre and post intervention assessment compared with the wait-control group.

Participants would be assessed on a case-by-case basis if they were in danger of suicide or doing harm to others. This will be defined by demonstrating actively suicidal or homicidal thoughts. At that point, the counselors will work with their supervisors to seek more specific psychiatric assistance with the individual.

12) Describe what happens to participants receiving therapy when the study ends or if a subject’s participation ends prematurely.

At the end of the study those counselors providing interventions found to be effective will continue to provide treatment, both to those currently receiving the treatment and to new clients. Persons who have not yet received the new intervention will be eligible to receive it regardless of whether they had previously received any treatments. If a subject’s participation ends prematurely for whatever reason, they could continue to work with a clinician or other care services as needed.

Any participant who does not come to a regularly scheduled session will be followed up by the counselor to determine whether they are a formal withdrawal or if there is some other barrier that can be overcome to continue treatment. If the participant does not want to participate further, the counselor or project staff will try to find out the reason for withdrawal. Otherwise, no further action will be taken. Our sample size calculations are based on an assumption that about 25% of participants will choose to withdraw from the study and from treatment.

13) Describe the process for referring subjects to care outside the study, if needed.

One local counterpart, Mae Tao Clinic, operates a primary care clinic with a Counseling Center and with referrals to Mae Sot Hospital, a Thai hospital in Mae Sot with limited in-patient psychiatric services. Clinical supervisors for the CBI intervention will be in weekly contact with JHU and consulting CBI experts on a routine basis and will communicate with them on an as needed basis. Persons who are found at screening or during the study to be in active danger of harming themselves or others will be immediately discussed with clinical supervisors and the safety response team for referral to the nearest and most appropriate treatment site.

14) For studies that evaluate interventions, have a randomized study design, and/or are a clinical trial, provide power calculations for projected sample size.

To calculate sample size, we relied on a pair-wise comparison – that is comparing the intervention to the wait-control condition. We calculated sample size and power calculations using effect size analyses of depression and trauma symptom scores. We estimated that to find a moderate effect size (.50) for either depression or trauma, we would need 120 participants in each arm. We used power=0.80, alpha=0.05, a design effect of 1.5, a drop-out rate of 25%, and a 1:1 ratio of intervention (case) to wait-control.

Based on our experience of both client and counselor drop-out and other challenges (i.e. people moving), we chose to inflate the recruitment sample size needed for each arm to 150, resulting in 300 participants. Given the rates of depression and trauma symptomatology found during the previous validity study, we estimate needing to screen approximately 400 torture survivors to identify 300 eligible and willing participants.

15) *Describe any plan for reporting test results to participants.*

After the intake interview is complete, the counselors will review the results of the depression symptoms subscale (Questions 3.1 – 3.17 in the screening/assessment instrument) and the trauma symptoms subscale (Questions 4.1 – 4.30) and will share these with the client. Data from the other scales will not be available to the clients at the time of the intake screening.

At the completion of the study, all study participants, including wait-controls at that time, will be told what the general results were and informed as to their options for receiving continuing or alternative forms of treatment.

5. Data Security and Protection of Subject Confidentiality

a. Are you applying for a Certificate of Confidentiality? No**.**

b. *Identify the data security plan below that best describes how you will minimize the risk of a breach of confidentiality by checking one of the boxes on the left side of this chart. If your study includes sequential phases that require different procedures, or does not fit these categories, explain in "Other".*

Note: Identifiers include name, address, SSN, hospital record number, etc., and other indirect identifiers (e.g., date of birth) that, when combined with other variables, may make a subject identifiable. These categories reflect minimal standards; you may impose more stringent protections. See the JHSPH Data Security Guidance at www.jhsph.edu/irb > Policies & Guidance > Guidance for additional information regarding best practices.

**Hard Copy of data collection form**: **Indicate your choice but typing an X in the appropriate box on the left:**	
	Hard copies of data collection materials have identifiers and are locked in a secure cabinet or room with limited access by specified individuals. When possible, redacted (de-identified) versions of the data collection sheets will be used for coding and analysis.
X	Hard copies of data collection materials include an ID code and do not have personal identifiers. However, a code linking the data to the subject's personal information is stored separately from the data collection sheets, and is locked in a secure cabinet or room with limited access by authorized individuals.
	Data are not collected on paper.
	Other (describe):
**Electronic Databases: Indicate your choice by typing an X in the appropriate box on the left: :** *Note: A de-identified version of the database should be used for data analysis except in instances in which identifying information is prerequisite for coding or analysis. Databases that retain identifying information require a higher degree of electronic security.*	
	The study is minimal risk and data collected are not sensitive in nature. No personal identifiers are included in the electronic database. Any electronic documents that link IDs to identifying information are stored on a computer in accordance with JHSPH Data Security guidance.
	Personal identifiers are included in the database. The data are stored on a computer that is password protected with a secure server. Transfer or storage on portable devices (e.g., laptops, flashdrives) is encrypted. The devices on which this information is stored are accessible only to individuals who need access to these data.
	No personal identifiers are included in the database but linkable identifiers exist separately and the data are sensitive in nature *(e.g., substance use, mental health, genetic propensities, sexual*

	practices or activities) such that disclosure could provide a risk to the individual. The codes are stored on a computer that is password protected with a secure server. Transfer or storage on portable devices (e.g., laptops, flashdrives) is encrypted. The devices on which this information is stored are accessible only to individuals who need access to these data.
X	No personal identifiers are included in the database but linkable identifiers exist separately and the data are sensitive in nature *(e.g., substance use, mental health, genetic propensities, sexual practices or activities*) such that disclosure could provide a risk to the individual. Information linking personal identifiers to ID codes of subjects entered into the database is stored separately on paper and locked away.

c. *If you are using participants' personal identifiers, describe any plans for disposing of identifiers including if, when and how that will be done.*

As part of a client monitoring process, the client's name, address and contact information will be maintained by the counselors and kept separate from study forms. For purposes of all data collection being carried out by counselors (pre- and post-intervention assessments and client monitoring forms) an assigned client ID number will be used. The Project Director, Field Project Coordinator, and Field Project Manager will keep a list of all the names, contact information and ID numbers. Clinical supervisors and counselors will have names, contact information and ID numbers only for the clients with whom they are working directly. The master list and all of the assessment forms will be kept securely under lock and key with access only by the research team.

Collection of identifiers is necessary to allow the counselors to keep track of their clients and in order to link the results of the screening interview with the repeat interview done after the intervention is completed. Only by having the personal identifiers linked to the code can we ensure that both interviews are with the same person. This is also necessary in that we do not plan to have the repeat interview carried out by the counselor providing the intervention to a given client but by another counselor participating in the study. At the end of the study (after completion of analysis comparing baseline and folllow-up results) the lists that the supervisors made that connect study forms with client information will be destroyed. All other forms will be kept per local organizational protocols for their own service files.

d. *Describe any plans for destroying data including if, when and how that will be done.*

The original screening and intake/assessment instruments will be kept in the BBP office in Mae Sot. Electronic records will be stored on a single, secure computer and on the PI and co-PI's computers and on a separate hard drive. Data from the baseline and follow-up interviews as well as some tracking data of client participation will be transferred to JHU via encrypted data files and stored at JHU in a password-protected computer in a locked office, Electronic copies of data will not be destroyed.

6. Recruitment process:

a. *Describe how participants will be recruited.*

As with the previous qualitative and validity studies, we will work with the local counterpart organizations to encourage members to present themselves for screening. Persons who are otherwise seen by counselors and other organization staff-members during their regular work will also be encouraged to provide their contact information for follow up.

b. *Explain how your recruitment materials will be used.*

Local partner organizations (BBP, AAPP, MTC and SAW) have developed a one-page handout and pamphlet in the local language (Burmese) that provides a brief description of the project and contact information for persons in the project whom they can contact for more information. These contact persons with the local partner organizations will then collect names and contact information for those who express interest in possibly taking part in the study..

c. *If relevant, address any privacy concerns associated with the recruitment process.*

Based on our previous studies using the same approach, we do not have any privacy concerns for the recruitment process as this step will involve conversations with program providers known and trusted in the community. Staff-members from the participating organizations have been made aware throughout the course of the study that they are not to share publicly any information about individuals who may contact them out of interest in participating in the study and in the CBI intervention.

7. Consent process and documentation:

a. *Describe who will obtain informed consent from participants, and how, when and where consent will be obtained.*

Prior to the screening process and intake interview, the counselor will verbally consent participants, informing them of the interview process, the intervention therapy, the re-interview process and the possible risks and benefits of participation. The interview will take place at a private location convenient to the potential client and could include the BBP, AAPP, SAW, or MTC offices or a private home. At the beginning of the consent process, the counselor will establish that the person meets eligibility criteria for inclusions—that is, they are 18 years or older, a Burmese national, and resident in the Mae Sot area. During the oral consent process, the potential client will be informed that they will first be asked a series of screening questions to assess their mental health symptomology. If the person is determined to be eligible for inclusion in the intervention study on the basis of these higher symptom scores, they will be told that they will be randomized into a case group or wait-control group and informed that they will either start the intervention within approximately one week or will be asked to wait several months. Following the intervention, both groups will be re-interviewed.

Thus the screening consent form will offer a recruited individual to consent to be screened for trauma exposure and for depression and trauma symptoms only. If the individual is deemed eligible for inclusion, the study consent form will offer the potential client an opportunity to complete the intake/assessment interview and to be randomized into a case or wait-control group for treatment (for the case group), and a follow-up interview several months later. For those in the wait-control group, they will be further asked if they are willing to be contacted once a month by the counselor while they are waiting for the follow-up interview.

Failure to show up for first session will disenroll a person from the study; we do not regard a client as fully consented unless they show up for Session 1 as they may have changed their mind after discussing participation with family members. If a client clearly (in the view of the counselor, clinical supervisor and study team) does not have mental capacity to give consent, then that person would not be asked to be in the study.

b. *If the study will involve vulnerable populations (e.g., children, prisoners, cognitively impaired adults, non-English-speakers, etc.) describe efforts to ensure their understanding*

of the research and the extra protections that will be in place to ensure their voluntary participation.

No specific vulnerable populations (beyond the designation of survivors of torture) will be enlisted in the study. All intervention materials and counseling sessions will be in Burmese.

c. *If a waiver of consent or a waiver or alteration of signed consent is requested, provide a justification for the waiver/alteration, and describe any alternate procedures for informing participants about the research.*

A waiver of signed consent is being requested. Given the sensitive nature of signing documents among this population, we will have the counselors read an oral consent script, both for the screening interview and, if eligible, for study participation. Whether the client gives verbal consent or declines, the counselors will sign the consent script form, indicating the client's decision; this form will then be kept on file for the duration of the study.

8. Risks:

a. *Describe the risks associated with the study and its procedures, including physical, psychological, emotional, social, legal, or economic risks.*

There is some risk that persons completing the screening and/or intake/assessment forms may be reminded of past traumatic events and become distressed. The CBI therapy includes discussion and recollection of the traumatic exposure with management of the emotional reactions with the counselor. Research and experience in the West suggests that exposure-based therapies are likely to be effective when treating individuals with post-trauma syndromes. There is some risk that such exposure could be harmful if the reactions are not properly managed. However, the emphasis of the training is on correct management, so that the result should be symptomatic relief. We do not expect any social, legal or economic risks. The identity of torture survivors is well known in the community and some belong to a well-known prisoner association. Assessment and treatment will be organized so as not to interfere with employment or other support activities.

b. *Describe steps to be taken to minimize those risks.*

If persons experience distress while completing the screening interview or the intake/assessment form they will be asked to notify the counselor and are free to pause or stop the assessment. If they remain distressed the counselor will provide counseling based on their existing skills and knowledge, and will offer to see the person again in a day or two for further treatment. With regards to CBI, the training emphasizes the management of reactions to exposure, so that the exposure should result in improvement and relief.

For all interventions, during each treatment session the current state of the client's symptoms is assessed. If indicated, the client will be referred by the counselor for assessment by the clinical supervisor, and, if indicated further, be treated by them or referred to a local facility for further assessment and treatment. The clinical supervisors will meet with the counselors weekly to monitor the progress of the program and how things are going with each client. To monitor the individual clients, the counselors will fill out session monitoring forms (see attachments) for each client after each session. As part of these forms, the counselors will record any changes in symptoms and problems that the client has. These forms, together with the weekly supervisory discussions, will form the basis for monitoring the current state of participants' symptoms and will be able to detect if symptoms worsen and the client needs additional services.

The Safety Plan that has been established for the intervention (and modified from a plan that has been used in Iraq) involves the counselor at each weekly session asking the client a series of questions, first explaining what—"I'm going to ask you some questions about safety"—and then why—""We ask every person these questions often because we want to be sure you're safe." The four questions are"

1. "Do you think about killing yourself"
2. "Do you have a plan to kill yourself?"
3. "Do you have a way to carry out this plan?"
4. "Have you ever tried killing yourself?"

If the client responds "yes" to any of the questions, the counselor should call their supervisor immediately after the session. If a client responds "yes" to either of the last 2 questions, the counselor is to call his/her supervisor immediately with the client still in the room. The counselor will ask the client to do their "safety work:" "We want to make sure you are safe. I know this might be hard. Can you give me your word that you will keep yourself safe for a short period of time - just over the next day?" The counselor will then encourage the client to set up a safety watch: "We want to help you keep yourself safe. Many times we use family members or friends to do this. Can you help me think of who in your family or which of your friends can be around? Can we work together to bring these people in to agree to help be with you so that you are safe?" The counselor and clinical supervisor will consult with the safety response team to determine appropriate next steps for care and treatment.

The safety response team on the ground comprises the counselor, the clinical supervisor, Dr. Htin Zaw, MD, (a clinical supervisor, and a member of the study team, and medical advisor to SAW), the organizational supervisor (there is one from each organization including Lae Lae from BBP) and Dr. Alice Khin from the Community Consultation Team. The team would consult amongst themselves and also contact Cate Lee, JHU Project Director in the field. She would in turn inform the JHU team, including the P.I. and Laura Murray, PhD (JHU faculty, a co-investigator and a psychologist) before determining the most appropriate course of action for a client. Options could include having Dr. Htin Zaw manage the case, asking Mae Tao Clinic (which has a Counseling Center and in-patient services) to manage the case, or referring the case to Mae Sot Hospital. Female clients may also be referred to SAW for temporary residence in one of their shelters and safe houses.

c. *Describe the research burden for participants, including time, inconvenience, out-of pocket costs, etc.*

For those who participate only in the screening interview, the burden would involve a 20-minute interview. Given that persons recruited for the initial screening interview would be assisted with transportation to the interview site, we do not anticipate that they would incur any out-of-pocket costs. Among those enrolled in the study, for the case group, the burden will be a one-hour initial assessment interview, one hour a week sessions for approximately 10 weeks of CBI therapy (including the time to the counseling site), and a one-hour post-intervention assessment. For the wait-control group, the burden will involve two one-hour assessment interviews and three to four monthly contacts while the intervention is being provided to the case group clients. Given that all of these services are in Mae Sot or nearby communities, the travel time required to get to them are minimal. As noted previously, local transportation costs will be reimbursed for study participants.

d. *Describe how participant privacy will be protected during data collection if sensitive questions are included in interviews.*

Counselor-subject interactions, including the initial screening and intake interviews and subsequent counseling sessions, will be carried out in private locations, including the BBP, SAW, AAPP, or MTC offices, with preference given to the BBP office which has three private counseling rooms available.

9. Benefits:

a. *Describe any potential direct benefits to participants from participating in the research (not including payment for participation).*

Our collaborative plan with partner organizations will result in all persons identified with significant trauma or depression symptoms receiving CBI treatment if it is found to be effective. Therefore, at the end of the study, those still suffering from significant symptoms that have not yet received the intervention will be eligible to receive it at that point in time.

b. *Describe potential societal benefits likely to derive from the research.*

Previous research has suggested that torture survivors feel (and are perceived by others to be) a burden to their families and communities because of reduced functioning. Our intention is that one or more of these interventions will improve social, emotional, cognitive and physical functioning and enable them to be more active and contributing members of their community. This could reduce the burden on families and society while improving their status within society.

10. Payment:

a. *Describe the form, amount, and schedule of payment to participants.*

We will provide financial assistance to cover any transportation costs for study participation where needed. Otherwise no payments will be made.

b. *Include the possible total remuneration and any consequences for not completing all phases of the research.*

Payments will be made to cover costs only, so there is no predicted total remuneration. There are also no consequences for non-completion (we would not be asking for the cost reimbursements to be repaid).

11. Safety monitoring:

a. *Describe how participant safety will be monitored, by whom, and how often.*

During each treatment session for the intervention, the level of symptom severity will be assessed by the counselor (see the example treatment monitoring form included under miscellaneous documents). If symptoms worsen the supervisor will be informed and the person, if indicated, will be either treated by a local clinically-trained counselor or referred to Mae Tao Clinic or Mae Sot Hospital for further assessment and treatment.

Independent safety monitoring will also be carried out by the studys Community Consultation Team (CCT), which comprises Burmese clinicians and service providers with counseling experience. The CCT serves in the capacity of local IRB for the project and includes among its members, Dr. Alice Khin, a member of the Burma Medical Association. The CCT will be regularly consulted as part of safety monitoring and will be notified in any event requiring activation of a client safety plan during the course of the study.

b. *Describe plans for interim analysis and stopping rules.*

We are not planning to establish a DSMB. Instead, client risk will be monitored at the individual level as in #11a above.

12. **Plan for reporting unanticipated problems/adverse events**:
Describe plan for reporting to the IRB and (if applicable) to the sponsor. Include plan for government-mandated reporting of abuse or illegal activity.

All of the counselors will be supervised weekly by local program and clinical supervisor staff. The supervisors will also be in weekly contact with the CBI trainers/experts in the US. Moreover, the study team in the field, will be in regular contact with the PI. Any unanticipated problems or adverse events will be reported to the local and the JHU IRB, along with the measures taken and the results. We do not anticipate that this study will result in the reporting of abuse or illegal activity, since the assessment instruments refer only to mental health symptoms and functioning. We will not be asking the migration status (possibly undocumented) of any of the participating Burmese populations.

13. **Other IRBs/Ethics Review Boards:**

As in previous phases of the study, the research was reviewed and approved by a local IRB: a Community Consultation Team established through the project and in consultation with the partner organizations with members of the team including local organizations not currently participating in this project. This committee does not have an FWA.

14. **Outside collaborations:**

The local collaborating organizations in this study are Burma Border Projects, Assistance Association for Political Prisoners, Social Action for Women, and Mae Tao Clinic.

15. **Oversight plan for student studies:**

This is not a student study though it will involve a PhD student, Cate Lee (Department of International Health) as field director and student investigator. Cate Lee has lived and worked on the Thai-Burma border for more than seven years and speaks Burmese and Thai. She and the JHU P.I. will maintain continuous contact throughout the fieldwork period through in person meetings and regular e-mail and telephone conversations. The P.I., Courtland Robinson, has direct oversight of the student investigators and will be in the field to provide training in human subjects research and begin the intervention study. The P.I. will monitor the field work process, and monitor ongoing study data collection and participate in data analysis.

16. **Oversight plan for studies conducted at non-JHSPH sites, including international venues, for which the JHSPH investigator is the responsible PI:**

Courtland Robinson, the study PI, will be on site to coordinate the training in study and consent procedures as well as setting up the study itself. He will be working closely with Cate Lee, the Project Director in Mae Sot as well as with other local members of the study team and with the JHU trainers. All clinical supervisors and study staff will complete the NIH-required training online. The counselors will receive training on human subjects' ethics and protection from the JHU study investigators. Interviewers will be provided with several days of intensive training prior to the start of the research phase. Training topics will include human subjects protection, interviewing techniques, and adherence to the study protocol. Human subjects protection training will occur during a one-day session. During the sessions, JHU and local supervisory staff will introduce research ethics themes and instruct interviewers on the necessity of and instructions on ensuring confidentiality and obtaining informed consent, demonstrating respect for the respondent, understanding the risks and benefits of the study, special interviewing techniques and ethical

considerations for working with survivors of torture and systematic violence, potential adverse events and how to handle them, and protocols for referral to local services. Following the training, interviewers will practice the consent process with their peers.
